# Textures of Personality: The Role of Attachment Insecurities and Defense Mechanisms in Maladaptive Personality Functioning

**DOI:** 10.3390/bs15091154

**Published:** 2025-08-25

**Authors:** Alessandro Vito Di Caro, Josephin Cavallo, Andrea Scalone, Alessia Passanisi, Adriano Schimmenti

**Affiliations:** 1 Department of Human and Social Sciences, UKE—Kore University of Enna, 94100 Enna, Italy; alessandrovito.dicaro@unikorestudent.it (A.V.D.C.); alessia.passanisi@unikore.it (A.P.); adriano.schimmenti@unikore.it (A.S.); 2 Department of Health Sciences, University of Florence, 50100 Florence, Italy; andrea.scalone@unikorestudent.it

**Keywords:** alternative model for personality disorders, attachment, defense mechanisms, personality

## Abstract

Attachment styles and defense mechanisms are widely recognized as central components in personality development. However, few empirical studies have examined their combined contribution to maladaptive personality traits within the dimensional framework of the DSM-5 Alternative Model for Personality Disorders (AMPD). This study investigated the extent to which adult attachment styles and defense mechanisms predict the five AMPD maladaptive personality domains: negative affectivity, detachment, antagonism, disinhibition, and psychoticism. Data were collected from a community sample of 400 adults (190 males, 47.5%), aged 18 to 69 years (M = 36.96; SD = 11.59). Hierarchical multiple regression analyses were conducted for each maladaptive personality domain to examine the predictive roles of attachment styles and defense mechanisms. Our findings indicate that each maladaptive personality domain is associated with specific configurations of attachment styles and defense mechanisms. In conclusion, the findings suggest the relevance of assessing adult attachment styles and defensive functioning in clinical contexts in order to deepen the understanding of the individuals’ personality profiles.

## 1. Introduction

Personality can be defined as a relatively stable set of psychological and behavioral traits and characteristics that contribute to shaping how individuals relate to their internal world and external reality (American Psychological Association, 2018). Historically, psychiatric classification systems have conceptualized personality pathology in a dichotomous manner; until the fourth edition of the Diagnostic and Statistical Manual of Mental Disorders, text revised (DSM-IV-TR; American Psychiatric Association [APA], 2000), clinicians could only determine the presence or absence of a personality disorder. The introduction of the DSM-5 (APA, 2013), alongside the categorical classification system, proposed a dimensional model, the Alternative Model for Personality Disorders (AMPD). This model evaluates personality functioning through dimensions of identity, self-direction, empathy, and intimacy (Criterion A) while also assessing five broad maladaptive personality trait domains, namely, negative affectivity, antagonism, disinhibition, detachment, and psychoticism (Criterion B). According to the AMPD, negative affectivity refers to a pervasive tendency to experience a broad spectrum of negative emotions with marked frequency and intensity. Individuals high in this trait may frequently report feelings such as anxiety, sadness, guilt, shame, worry, or anger (APA, 2022). These emotional states are often accompanied by a characteristic cluster of behaviors and interpersonal functioning deficits, including tendencies toward self-directed harm and excessive dependence on others (Watson & Clark, 1984, 2020). Antagonism captures a pattern of behaviors that place individuals in opposition to others, including traits such as inflated self-importance, a persistent expectation of preferential treatment, and a marked disregard for others’ needs or feelings (APA, 2022). Individuals high in antagonism may exhibit a lack of empathy and a willingness to exploit interpersonal relationships in ways that serve their own self-interest (Ball Cooper et al., 2021; Ro et al., 2017). Detachment is defined as an avoidance of social and emotional engagement, as well as a general withdrawal from interpersonal relationships, ranging from limited participation in everyday social interactions to the absence of close friendships and intimate bonds (APA, 2022). Individuals who display high detachment also show restricted affective expression, with diminished capacity to experience or convey positive emotions and a general blunting of hedonic responsiveness (Ball Cooper et al., 2021; Krueger et al., 2012a). Disinhibition reflects a tendency to act on impulses and seek immediate gratification without adequately considering the consequences of one’s actions (APA, 2022). Individuals high in disinhibition are more likely to be driven by momentary thoughts, emotions, or environmental stimuli, often disregarding previous learning or long-term goals (Clark & Watson, 2008; Mullins-Sweatt et al., 2019). Psychoticism encompasses a variety of unusual, eccentric, or culturally deviant behaviors and cognitions (APA, 2022). This trait may manifest through disturbances in thought processes—such as perceptual anomalies and dissociative experiences—as well as through atypical beliefs or ideas that diverge markedly from conventional norms (Ball Cooper et al., 2021; Gervasi et al., 2017). Building on the AMPD perspective, recent years have seen growing interest in dimensional approaches to personality assessment, as they address key limitations of categorial classification, such as high comorbidity rates and poor discriminant validity (Mulder, 2024).

A substantial body of research highlights the role of early relational experiences in shaping personality development (Ainsworth et al., 1978; Bowlby, 1988; Lorenzini & Fonagy, 2013; Parker et al., 1999; Skabeikyte-Norkiene et al., 2022). Bowlby’s attachment theory (Bowlby, 1969) provides a foundational framework for understanding how early interactions with caregivers influence adult personality structures. Attachment patterns established in infancy significantly contribute to the development of core characteristics of personality, such as affect regulation, stress management, attentional control, mentalization, and self-agency (Cooke et al., 2019; Fonagy, 2010; Luyten & Fonagy, 2014; Nunes et al., 2022; Pallini et al., 2019; Santoro et al., 2021; Vondra et al., 2001). Attachment is an innate motivational system that prompts children to seek proximity to their caregivers when experiencing stress or perceived threat. The quality of caregiver interactions and responsiveness shapes the development of internal working models of attachment, which influence self-perception, interpersonal relationships, and emotional regulation throughout life (Fonagy et al., 2002; Main & Solomon, 1990).

Building on this perspective, Bartholomew and Horowitz (1991) identified four primary adult attachment styles: secure, dismissing, preoccupied, and fearful. Secure attachment, characterized by positive representations of both self and others, supports stable emotional regulation and interpersonal functioning (Nolte et al., 2011). In contrast, dismissing individuals, who hold a positive view of the self but a negative view of others, often lacked consistent emotional closeness with their caregivers during childhood, which resulted in a defensive deactivation of their attachment system, leading them to devalue attachment and suppressing attachment-related distress (Bartholomew & Horowitz, 1991; Bennett, 2005; Mikulincer & Shaver, 2016). Preoccupied individuals, characterized by a negative self-representation but a positive view of others, were typically exposed to caregiving marked by emotional unpredictability; thus, they tend to exhibit heightened sensitivity to perceived threats and a tendency toward compulsive care-seeking and dependency (Bartholomew & Horowitz, 1991; Botbol, 2010; Mikulincer & Shaver, 2016). Fearful individuals—characterized by negative representations of both their self and others—often experienced caregiving that was either frightened or frightening and marked by significant caregivers’ failures in attuning to the child’s emotional needs (Bartholomew & Horowitz, 1991; Main & Hesse, 1990). These individuals are at increased risk for emotion dysregulation and difficulties in reflective functioning (Fonagy & Target, 2005; Santoro et al., 2021; Slade et al., 2005).

In fact, it is noteworthy that a persistent lack of trust in interpersonal relationships can significantly impair an individual’s capacity for mentalization, emotion regulation, and adaptive learning from experience. Deficits in these areas have been linked to difficulties in affect modulation and social functioning, underscoring the long-term impact of insecure attachment patterns. In particular, attachment vulnerabilities, especially in the context of early maltreatment experiences, can contribute to the development of psychopathology (Midolo et al., 2020) and have also been linked to an increased vulnerability to personality disorders (Levy et al., 2015; Lorenzini & Fonagy, 2013; Pad et al., 2022; Siczek & Cieciuch, 2024; Smith & South, 2024). For example, insecure attachment styles have been associated with core features of borderline personality disorder, such as heightened negative affectivity and impulsivity (Scott et al., 2009). Other studies suggest that individuals with personality disorders frequently exhibit attachment-related difficulties that contribute to maladaptive emotion regulation and interpersonal difficulties (Aaronson et al., 2006; Brennan & Shaver, 1998; Buchheim & Diamond, 2018; Erkoreka et al., 2022; Luyten & Fonagy, 2014).

Since attachment, as conceptualized by Bowlby (1969, 1973, 1980), constitutes a fundamental motivational system aimed at maximizing a child’s chances of survival, the early caregiving environment plays a crucial role in shaping the internal working models of attachment. Within this context, children must develop ways to protect themselves from both internal conflict and external threats. It is within these early relational experiences that defense mechanisms begin to emerge. Defense mechanisms are predominantly automatic psychological responses to internal or external stressors, aimed at managing unbearable affects, wishes, impulses, thoughts, and memories, thereby protecting the individual from emotional conflict (Lingiardi & McWilliams, 2017). Rooted in psychoanalytic theory, defense mechanisms are understood as potentially adaptive processes that vary depending on the developmental stage and contextual demands (Cramer, 2006, 2015). In line with the model proposed by Vaillant (1971), Andrews et al. (1989) suggested that defense mechanisms can be grouped into three overarching styles: mature, neurotic, and immature. While mature defenses (e.g., sublimation) are associated with adaptive functioning and the modulation of emotions without significantly distorting reality, neurotic defenses (e.g., undoing) involve only a partial acknowledgment of internal conflict and a reworking of its emotional impact through an internal modification of the information. In contrast, immature defenses (e.g., splitting) serve to deny the existence of conflict or to externalize responsibility (Andrews et al., 1989). Persistent reliance on immature defense mechanisms is often associated with lower personality functioning and greater psychopathological risk (Benítez Camacho et al., 2010; Di Giuseppe et al., 2020a; Gioia et al., 2023; Kernberg, 1975; Perry & Cooper, 1989). A concise definition of each defense mechanism discussed in this article is provided in Table 1.

The relationship between defense mechanisms and personality traits has been widely investigated. Previous research indicates that immature defense mechanisms—such as acting out, projection, and splitting—are associated with increased vulnerability to maladaptive personality domains, whereas mature defenses are linked to greater psychological resilience and adaptability (Granieri et al., 2017). Similarly, Strandholm et al. (2016) showed that adolescents exhibiting neurotic and immature defense styles are at increased risk of developing personality disorders in adulthood, with specific mechanisms such as displacement, isolation, and reaction formation emerging as independent predictors. In sum, more mature defense mechanisms have been associated with healthier personality functioning, whereas more pathological personality profiles tend to rely on immature and rigid defenses (Di Giuseppe et al., 2020b), thereby supporting the notion that defense mechanisms play a significant role in shaping personality outcomes. To the best of our knowledge, no empirical studies have yet integrated these constructs within the DSM-5 AMPD framework. This study aims to fill this gap by investigating how attachment styles and defense mechanisms predict distinct maladaptive personality domains.

## 2. Materials and Methods

### 2.1. Participants and Procedure

The study sample comprised 400 community-dwelling adults from Italy (190 males, 47.5%), aged between 18 and 69 years (M = 36.96; SD = 11.59). The average number of years of education was 16.65 (SD = 4.52). Participants were recruited via advertisements on social media platforms (e.g., Facebook) and provided informed consent prior to participation. Each participant completed a sociodemographic schedule and self-report measures.

### 2.2. Measures

The Relationship Questionnaire (RQ; Bartholomew & Horowitz, 1991; Italian translation by Carli, 1995) is a self-report measure used to assess four prototypical adult attachment styles: secure, dismissing, preoccupied, and fearful. Each attachment style is expressed through a first-person statement. An example item is “I am comfortable without close emotional relationships. It is very important to me to feel independent and self-sufficient, and I prefer not to depend on others or have others depend on me”—a description of the dismissing attachment style. Participants are asked to rate their level of agreement with each statement using a 7-point Likert scale (1 = Strongly disagree; 7 = Strongly agree), providing a dimensional assessment of attachment styles. The RQ demonstrated both discriminant validity (Griffin & Bartholomew, 1994) and test–retest reliability (Scharfe & Bartholomew, 1994).

The Defense Style Questionaire-40 (DSQ-40; Andrews et al., 1993; Italian validation by Farma & Cortinovis, 2000) is a 40-item self-report questionnaire designed to assess twenty different defense mechanisms grouped into three distinct defensive styles: mature (sublimation, humor, anticipation, and suppression), neurotic (undoing, pseudo-altruism, idealization, and reaction formation), and immature (projection, passive aggression, acting out, isolation, devaluation, autistic fantasy, denial, displacement, dissociation, splitting, rationalization, somatization). An example item is “People tend to mistreat me” (projection). Participants are asked to rate their level of agreement with each statement using a Likert scale from 1 (“totally disagree”) to 9 (“totally agree”). In the current study, the Cronbach’s alpha coefficients were 0.77 for mature style, 0.74 for neurotic style, and 0.89 for immature style.

The Personality Inventory for DSM-5-Brief Form-Adult (PID-5-BF; Krueger et al., 2012b; Italian validation by Fossati et al., 2015) consists of 25 items designed to assess five domains of maladaptive personality traits (negative affectivity, detachment, antagonism, disinhibition, and psychoticism). An example item is “I get irritated easily by all sorts of things” (related to the domain of negative affectivity). Answers are marked on a 4-point Likert scale ranging from 0 (very false or often false) to 3 (very true or often true). In the present study, the Cronbach’s α coefficients were 0.66 for negative affectivity, 0.70 for detachment, 0.57 for antagonism, 0.68 for disinhibition, and 0.78 for psychoticism. Therefore, while most personality domains demonstrated sufficient levels of internal consistency, the Cronbach’s α for antagonism was barely acceptable. Although previous research (Fossati et al., 2013; Al-Dajani et al., 2016) has reported satisfactory reliability estimates for this domain, it is possible that its conceptual heterogeneity (encompassing distinct traits such as manipulativeness, deceitfulness, and grandiosity) may contribute to greater psychometric instability, with relatively low alpha coefficients likely reflecting both the limited number of items and the multidimensional nature of the construct (Schweizer et al., 2025).

### 2.3. Statistical Analysis

Descriptive statistics were calculated for all variables included in the study. Associations between PID-5 trait domains and the other variables were examined through two separate Pearson’s *r* correlation analyses: one focusing on adult attachment styles and the other on defense mechanisms. Separate hierarchical multiple regression analyses were conducted for each of the five PID-5 trait domains, treated as dependent variables.

## 3. Results

Descriptive statistics are presented in Table 2. Male participants exhibited higher levels of secure and preoccupied attachment styles, as well as significantly greater use of the following defense mechanisms: anticipation, suppression, idealization, passive aggression, isolation, autistic fantasy, denial, and dissociation. Regarding personality trait domains, males also showed significantly higher levels of antagonism and disinhibition.

In contrast, female participants reported more years of education, higher levels of negative affectivity, and higher use of the defense mechanism of somatization.

Significant associations were identified through Pearson’s *r* correlations (see Table 3 and Table 4). The personality domain of negative affectivity was significantly and positively correlated with fearful attachment style and preoccupied attachment style. It was also positively correlated with the following defense mechanisms: anticipation, undoing, pseudo-altruism, idealization, reaction formation, projection, passive aggression, acting out, isolation, devaluation, autistic fantasy, denial, displacement, splitting, and somatization. In contrast, negative affectivity showed a significant and negative correlation with age, years of education, secure attachment style, and humor defense mechanism.

The personality domain of detachment showed significant and positive correlations with fearful attachment style, preoccupied attachment style, and dismissing attachment style. It was also positively correlated with anticipation, undoing, pseudo-altruism, idealization, projection, passive aggression, acting out, isolation, devaluation, autistic fantasy, denial, displacement, splitting, and somatization. Conversely, detachment was significantly and negatively correlated with years of education and secure attachment.

The personality domain of antagonism was positively correlated with fearful attachment style, preoccupied attachment style, and dismissing attachment style, as well as with the defenses of anticipation, suppression, undoing, pseudo-altruism, idealization, projection, passive aggression, acting out, isolation, devaluation, autistic fantasy, denial, displacement, dissociation, splitting, and somatization. Antagonism was also significantly and negatively correlated with years of education.

The personality domain of disinhibition was positively correlated with fearful attachment style, preoccupied attachment style, and dismissing attachment style, as well as with the following defenses: suppression, undoing, pseudo-altruism, idealization, projection, passive aggression, acting out, isolation, devaluation, autistic fantasy, denial, displacement, dissociation, splitting, and somatization. Disinhibition also showed significant and negative correlations with years of education and secure attachment style.

The personality domain of psychoticism was positively correlated with fearful, preoccupied, and dismissing attachment styles, as well as with the following defense mechanisms: sublimation, anticipation, suppression, undoing, pseudo-altruism, idealization, reaction formation, projection, passive aggression, acting out, isolation, devaluation, autistic fantasy, denial, displacement, dissociation, splitting, and somatization. Psychoticism was negatively and significantly correlated with age, years of education, and secure attachment style.

To evaluate the impact of attachment styles and defense mechanisms on the prediction of each personality domain of the PID-5, we conducted five hierarchical multiple regression analyses. Sociodemographic variables (gender, age, and years of education) were entered in Step 1 to control for their influence. Attachment styles were entered in Step 2, followed by defense mechanisms in Step 3.

The results of the first regression analysis, with negative affectivity as the dependent variable, are presented in Table 5. Sociodemographic variables (gender, age, and years of education) were significant predictors, accounting for 9.9% of the variance (Model 1). Adding attachment styles in the second step significantly increased the explained variance by an additional 12.6% (Model 2), and the inclusion of defense mechanisms further increased the explained variance by 30.1% (Model 3).

In the final model, explaining 52.6% of variance, the positive predictors of higher levels of negative affectivity included female gender and the following defense mechanisms: idealization, projection, acting out, autistic fantasy, and displacement. In contrast, negative predictors were age, secure attachment, dismissing attachment, and the defense mechanisms of humor and suppression.

The results of the second regression analysis, concerning the domain of detachment, are presented in Table 6. The sociodemographic variables (gender, age, and years of education) accounted for 5.4% of the variance, with only years of education emerging as a significant predictor in Model 1. Adding attachment styles in the second step significantly increased the explained variance by an additional 24.1% (Model 2). Finally, the inclusion of defense mechanisms further increased the explained variance by 19.4% (Model 3). In the final model, explaining 48.9% of the variance, the positive predictors of increased levels of detachment included fearful attachment, dismissing attachment, and the defense mechanisms of isolation and autistic fantasy; negative predictors were secure attachment and sublimation.

The results of the third regression analysis, with antagonism as the dependent variable, are presented in Table 7. Among the sociodemographic variables, only gender emerged as a significant predictor, with Model 1 accounting for 4.8% of the variance. Adding attachment styles in the second step increased the explained variance by only 3% (Model 2). Finally, the inclusion of defense mechanisms further increased the explained variance by 14.1% (Model 3). In this final model, explaining 21.9% of the variance, the positive predictors of increased levels of antagonism included the following defense mechanisms: projection, denial, and splitting. In contrast, negative predictors were male gender and reaction formation. Therefore, no significant effect emerged for attachment styles. However, considering that the internal consistency of the scale (α = 0.56) indicated limited reliability, these results should be interpreted with caution.

The results of the fourth regression analysis, concerning the domain of disinhibition, are presented in Table 8. In Model 1, which explained 10.7% of the variance, gender and years of education were both significant predictors. Adding attachment styles in the second step (Model 2) increased the explained variance by an additional 3.7%. Finally, adding defense mechanisms in Model 3 increased the explained variance by 27%. In this final model, explaining 41.4% of variance, the positive predictors of increased levels of disinhibition were acting out, dissociation, and splitting; negative predictors were years of education, secure attachment, and the following defense mechanisms: anticipation, devaluation, and rationalization.

The results of the last regression analysis, related to the domain of psychoticism, are presented in Table 9. In Model 1, the sociodemographic variables accounted for 9.1% of the variance, with both age and years of education entering as significant predictors; including attachment styles (Model 2) increased the explained variance by 7.5%; finally, adding defense mechanisms further increased the explained variance by 22.1% (Model 3). For this final model, explaining 38.8% of the variance, the positive predictors of increased levels of psychoticism included the defense mechanisms of undoing, projection, acting out, isolation, and autistic fantasy. In contrast, negative predictors were secure attachment, anticipation, and devaluation.

## 4. Discussion

The primary aim of the present study was to examine how specific adult attachment styles and defense mechanisms predict each of the five maladaptive personality trait domains outlined in the AMPD model. The following discussion considers the main findings of the study in light of this objective and their potential clinical implications.

### 4.1. Relationships Between Attachment Styles, Defense Mechanisms, and Maladaptive Personality Domains

When examining the correlations among the variables, most PID-5 domains were positively associated with attachment insecurity. Negative affectivity was positively associated with both fearful and preoccupied attachment styles. Conversely, all other domains—except antagonism—were negatively associated with secure attachment. These findings align with previous literature underscoring the association between maladaptive personality traits and insecure attachment (Pad et al., 2022; Siczek & Cieciuch, 2024; Smith & South, 2024). In this context, the only unexpected finding was the positive association between detachment and preoccupied attachment, as it contrasts with the model proposed by Bartholomew and Horowitz (1991), which characterizes preoccupied attachment by a hyperactivation of the attachment system (Santoro et al., 2021) and a tendency to seek closeness, rather than detachment. However, this result may reflect the negative self-model implied in preoccupied attachment, which could distort bivariate correlations but be accounted for in multivariate regression analyses. Additionally, all maladaptive personality domains demonstrated negative associations with years of education, suggesting a potential protective role of education against personality disorders. Negative affectivity and psychoticism were also negatively associated with age, in line with prior research documenting a decline in negative affectivity and risk for psychotic breakdowns across the lifespan (Charles et al., 2001; Mroczek & Kolarz, 1998; Shallcross et al., 2013).

With regard to defense mechanisms, all PID-5 trait domains showed predominantly positive associations with immature (e.g., projection) and neurotic (e.g., undoing) defenses. These findings are consistent with the existing literature (Anzani et al., 2018; Granieri et al., 2017; Muris et al., 2003), which highlights the link between maladaptive personality traits and the use of neurotic and immature defenses. However, some defense mechanisms traditionally classified as mature—such as anticipation, suppression, and sublimation—were also positively associated with certain maladaptive personality domains. Specifically, anticipation was linked to negative affectivity, detachment, antagonism, and psychoticism; suppression was linked to antagonism, disinhibition, and psychoticism; sublimation was linked to psychoticism. While these associations may appear inconsistent with conventional categorizations of defenses, an intriguing hypothesis could be that they reflect efforts to regulate or even conceal dysfunctional aspects of personality. For example, the positive association between anticipation and antagonism may suggest that even typically adaptive defenses can serve instrumental purposes, such as attempting to control others or strategically manage social interactions; this interpretation aligns with literature on psychopathy (Lynam & Miller, 2019; Spantidaki Kyriazi, 2021; Vachon, 2019), where interpersonal manipulation is a defining feature of antagonistic traits.

### 4.2. Predictive Models of Maladaptive Personality Functioning

When interpreting the results of hierarchical multiple regression analyses, it is important to consider them as complementary to bivariate correlations, rather than viewing them as inherently more meaningful. Bivariate correlation analyses revealed the basic, direct associations between pairs of variables, providing an overview of the typical relationships among attachment styles, defense mechanisms, and maladaptive personality domains. However, these associations do not account for the potential influence of other variables in the model. In contrast, hierarchical multiple regression analyses allowed for a fine-grained understanding of the relationship among the variables by examining how the potential predictors jointly explain variance in maladaptive personality domains while controlling for the effects of variables entered in earlier steps. This method helped identify broader patterns of maladaptive personality functioning, offering distinct and more integrative insights into how attachment styles and defense mechanisms contribute to maladaptive personality features. Therefore, the two approaches served different but complementary purposes; correlation provided a foundational map of relationships, whereas regression uncovered the structure and relative influence of these relationships within a multivariate context.

#### 4.2.1. Predictors of Negative Affectivity

Consistent with previous findings (Eaton et al., 2012; Gao et al., 2020; Kramer et al., 2008; Tung et al., 2018), our results support the notion that females, compared to males, are more likely to exhibit higher levels of negative affectivity and are thus at increased risk for developing internalizing disorders. Moreover, in line with the previous literature (Cooke et al., 2019), our findings indicate that individuals with secure attachment styles report lower levels of negative affectivity. This outcome is likely due to their enhanced capacity for emotion regulation, which enables more adaptive and effective management of negative emotional states (Carnelley et al., 2018; McGuire et al., 2018; Pereg & Mikulincer, 2004; Rowe et al., 2020). A negative predictive relationship was also found for dismissing attachment style, indicating that higher levels of avoidant attachment are associated with lower levels of negative affectivity. This result may be better understood in light of the core characteristics of this attachment style, which is defined by a pronounced tendency toward emotional disengagement and self-reliant emotion regulation in interpersonal contexts (Bartholomew & Horowitz, 1991). This affective disengagement strategy may limit the conscious processing of negative emotional states—especially those arising within interpersonal contexts—thereby contributing to reduced levels of negative affectivity (Bartholomew & Horowitz, 1991; Connors, 1997; Richardson et al., 2023). Regarding defense mechanisms, the findings indicate that higher levels of negative affectivity are positively predicted by mechanisms classified within the neurotic and immature categories. This aligns with recent research suggesting that elevated negative affectivity is linked to a greater reliance on less mature defensive mechanisms (Remmers et al., 2023). Defense mechanisms such as projection, acting out, and displacement may function as means through which individuals with elevated negative affectivity try to regulate emotions perceived as intolerable, notably anger or shame. Specifically, acting out entails the expression of these emotions via impulsive, externally directed behaviors intended to mitigate or obviate the awareness of distressing emotional experiences. In such instances, the intensity of affect may compromise reflective processing, impede cognitive function, and adversely affect decision making (Corruble et al., 2004; Frosch, 1977; Ponsi, 2017). Similarly, projection and displacement enable individuals to externalize distressing emotional experiences, thereby making them partially more manageable or transferring the burden of emotional processing onto interpersonal contexts. When such externally oriented defense mechanisms prove inadequate, individuals may retreat into autistic fantasy and idealization. The combination of these two mechanisms may lead to a withdrawal into the internal world, where individuals rely on idealized or salvific fantasies (such as being rescued by an external figure perceived as capable of meeting one’s emotional needs) as a means of protecting themselves from emotional disintegration or the onset of an acute depressive state (Corruble et al., 2004; McWilliams, 2011; Perry, 1990; Schimmenti et al., 2019). However, this process of idealization—rooted in a rigid, perfectionistic view of the other—can leave the individual particularly vulnerable to disappointment when such expectations are unmet. This disconfirmation may trigger an intense negative emotional response, such as anger or frustration, potentially leading to significant psychological distress (Garza-Guerrero, 2000). Accordingly, mature defenses such as humor and suppression negatively predict negative affectivity, suggesting that individuals with elevated levels of negative affectivity may struggle to engage in defenses that require a stable self-concept and adequate emotional regulation capacities (Kernberg, 1975; McWilliams, 2011; Wang et al., 2024). The presence of dysregulated emotional states, coupled with a predominant reliance on immature defenses, appears to reflect a less cohesive personality organization—one that limits the availability and effectiveness of more adaptive defensive processes.

#### 4.2.2. Predictors of Detachment

The findings concerning detachment are also consistent with the existing literature; detachment is a hallmark of schizoid and avoidant personality disorders (APA, 2022), both of which are characterized by a tendency to withdraw from interpersonal relationships (Beeney et al., 2017; Lingiardi et al., 2015; McWilliams, 2011). Within this framework, the predictive role of fearful and avoidant attachment styles aligns with the broader tendency of individuals high in detachment to avoid emotional closeness and to maintain low trust about the availability and reliability of others. Such attachment styles reflect internal representations of others as untrustworthy or threatening, prompting individuals to maintain emotional distance as a protective stance against relational experiences perceived as dangerous (Bartholomew & Horowitz, 1991). Conversely, securely attached individuals are less likely to develop a personality structure oriented toward detachment, as their internal representations of others tend to be more positive (Bartholomew & Horowitz, 1991) and, thus, less likely to be perceived as threatening. From this perspective, it is understandable that individuals with insecure attachment styles are more likely to rely on immature defense mechanisms that serve to distance them from relationships experienced as unpredictable or uncontrollable. Within this dynamic, isolation and withdrawal into autistic fantasy emerged as significant and positive predictors. Through isolation of affect, individuals can reduce the subjective experience of emotional vulnerability in interpersonal contexts (Bowins, 2004; McWilliams, 2011; Vaillant, 1992). In an effort to preserve the self, this process could reduce emotional engagement and maintain a cognitive focus on external events while disengaging from the corresponding affective states. At the same time, attention and imaginative investment may shift toward a more controllable inner world. Through autistic fantasy, this internal world becomes the privileged arena for mental activity—a psychic retreat that offers an illusory sense of self-sufficiency (Little, 2020; McWilliams, 2006, 2011). Within this internalized space, individuals construct imaginary scenarios that at least partially replace real engagement with the environment and with others (Schimmenti et al., 2019). This progressive disinvestment from interpersonal relationships, accompanied by withdrawal into the internal world, inhibits the individual’s capacity to derive pleasure from relationships and external activities. This is likely one of the reasons why our results indicate that individuals with high levels of detachment are less likely to rely on sublimation as a defense mechanism. When affect is isolated and split off from lived experience in favor of a predominantly cognitive focus, individuals are left without the emotional component of experience that is necessary for symbolic and creative transformation of internal distress; at the same time, the withdrawal into autistic fantasy fosters a regressive and self-referential mode of expression, which replaces investment in reality with a focus on the internal experience, further diverting resources away from the possibility of sublimating impulses through relational or culturally meaningful activities.

#### 4.2.3. Predictors of Antagonism

The observed gender difference in the antagonism domain aligns with previous research, which highlights a higher prevalence of narcissistic traits among males (Grijalva et al., 2015). Antagonism, in fact, is commonly observed in both narcissistic and antisocial personality structures (Miller & Lynam, 2019; Spantidaki Kyriazi, 2021).

Within this framework, the use of specific defense mechanisms may serve to preserve the individual’s inflated self-image and defend against internal vulnerability. Denial, for instance, may serve to uphold the individual’s inflated self-image by shielding them from potentially destabilizing feelings of shame and inadequacy that would otherwise reach consciousness, thereby preserving a sense of grandiosity (Kernberg, 1998). In personality organizations characterized by more primitive functioning, splitting supports a polarized view of the self and others, one that precludes the integration of ambivalent representations (McWilliams, 2011; Perry, 1990; Vaillant, 1992; Yeomans et al., 2015). Others may thus be perceived as entirely good when they serve the individual’s grandiosity or manifest goals but wholly bad and threatening when they do not (Caligor et al., 2015; Campbell et al., 2005; Lachowicz-Tabaczek et al., 2021). This process, in turn, facilitates and reinforces projection; given a split perception of others, they become ideal targets—or receptacles—for the externalization of affects or impulses that could threaten the grandiose self-image maintained by denial.

In the same vein, in personality configurations where antagonism constitutes a central organizational axis, it is less likely that individuals display the defense mechanism of reaction formation. Since reaction formation is a defense mechanism that involves managing emotional conflict by transforming unacceptable impulses into their opposite attitudes or behaviors (Perry, 1990), in individuals with high levels of antagonism, unacceptable impulses are not typically experienced as morally troubling or guilt-inducing but are more likely to be acted upon directly, without the need for defensive disguises (Lynam & Miller, 2019).

#### 4.2.4. Predictors of Disinhibition

Since disinhibition has been conceptually linked to both affect dysregulation and impaired interpersonal functioning (Gratz et al., 2009; Mostajabi & Wright, 2024), the identification of secure attachment as a negative predictor in the regression model suggests that difficulties in establishing and maintaining stable, trusting relationships—free from fears of abandonment or rejection—may increase vulnerability to disinhibited behavior. The inability to rely on secure attachment relationships may deprive individuals of an important regulatory function, thereby contributing to impulsivity and emotional instability. Our findings also indicate that disinhibition is associated with the deployment of immature defense mechanisms commonly observed in borderline personality structures (Nigg et al., 2005). Within this framework, the impulsive behaviors associated with acting out—together with the dichotomous perception of self and others characteristic of splitting, which impedes the integration of emotional states and internal representations—appear to contribute to the development of disorganized and poorly regulated behaviors, marked by deficits in emotional control and self-regulation.

The presence of dissociation may further amplify these vulnerabilities, as it can promote dysregulated behavioral patterns (Schimmenti & Caretti, 2016), which often manifest as impulsive and disinhibited actions. A substantial body of research has documented the co-occurrence of dissociation and impulsivity in individuals experiencing difficulties with emotional regulation, highlighting a high-risk profile for the emergence of dysfunctional behaviors and maladaptive outcomes such as self-harm, substance abuse, and interpersonal instability (Evren et al., 2013; Gori et al., 2016; Gratz, 2006; Somer, 2003; Ullman & Najdowski, 2009; Zlotnick et al., 1996, 1997). Taken together, these findings suggest that dissociation and impulsivity may interact synergistically to undermine adaptive defense mechanisms and behavioral control. Difficulties in emotional regulation may also help explain why anticipation and rationalization emerged as negative predictors of disinhibition in our study. The effective deployment of these mature defense mechanisms—both of which rely on higher-order cognitive functions such as foresight and reflective thinking—may be compromised in individuals experiencing affect dysregulation (Di Pierro et al., 2015). Engaging in rationalization requires a certain level of cognitive control and self-awareness (Cushman, 2020; Musslick & Cohen, 2021), while the use of anticipation requires the capacity to tolerate anticipated affect—capacities that are typically diminished in disinhibited individuals, who tend to act impulsively and with limited regulation. As a result, those who rely more on rationalization and anticipation are generally less prone to disinhibited behavior.

Finally, the presence of devaluation as a negative predictor of disinhibition may reflect the fact that disinhibited individuals, despite their relational difficulties, still need to rely on interpersonal bonds. Since devaluation entails attributing excessively negative qualities to others, its use might threaten the preservation of these bonds, which are nevertheless crucial in containing the potentially harmful consequences of their dysregulated behavior and in providing a temporary external anchor for emotional regulation.

#### 4.2.5. Predictors of Psychoticism

Psychoticism is commonly associated with personality disorders included in Cluster A, including paranoid, schizoid, and schizotypal personality disorders (APA, 2022). These disorders are marked by impairments in reality testing, eccentric behavior, and magical thinking (APA, 2022; Gabbard, 2014). In line with this framework, the identification of secure attachment as a negative predictor in our study suggests that individuals with high psychoticism may experience the interpersonal world as insufficiently safe or trustworthy, leading to a diminished capacity to rely on external sources for emotional regulation and relational engagement. Within this context, the positive association with autistic fantasy, projection, isolation, acting out, and undoing as defense mechanisms becomes more understandable. The use of autistic fantasy may emerge as a response to a weakened grasp of reality, prompting individuals to retreat into an internal world, protecting themselves from the terror of being engulfed or controlled by others (Schimmenti et al., 2019), while projecting distressing or disowned aspects of the self onto the external environment (McWilliams, 2011). This dynamic may foster a perception of the external world as inherently threatening, thereby promoting the use of isolation as a defensive mechanism and leading the individual to rely on bizarre and eccentric thinking in order to manage potentially unbearable emotions of terror and regain control. However, given that interaction with the external environment is unavoidable, the inability to regulate negative emotional states—sustained by bizarre beliefs about the outside world—may trigger the use of acting out as a defense mechanism (Gabbard, 2014). In response, the individual may subsequently engage in undoing, enacting compensatory behaviors aimed at reducing anxiety or guilt (Freud, 1937; Klein, 1946; McWilliams, 2011; Perry, 1990; Vaillant, 2000). The affective withdrawal from external reality may, in turn, account for the reduced use of defenses such as devaluation, which presuppose a minimal investment in the object for its value to be negated (Perry, 1990; Vaillant, 1992, 2000). Similarly, the difficulties with reality testing may compromise the individual’s ability to access more mature defenses, such as anticipation—a defense mechanism that requires the affective capacity to tolerate anticipatory anxiety and the cognitive ability to reflect on future outcomes (Perry, 1990; McWilliams, 2011).

### 4.3. Notes on Gender Differences

A note must be added on the findings concerning gender differences. In fact, the results of the present study revealed differences that, while partially consistent with the existing literature, also displayed some notable divergences. Regarding attachment styles, male participants reported significantly higher scores in both secure and preoccupied attachment. This finding partially contrasts with previous research, which generally suggests that women are more likely to exhibit preoccupied attachment patterns (Del Giudice, 2011). In terms of defense mechanisms, the findings confirmed gender-related differences consistent with prior studies (e.g., Cramer, 1991, 2002; Hibbard & Porcerelli, 1998), which suggest that gender influences both the preference for and frequency of specific defenses. Specifically, male participants showed a greater tendency to employ anticipation, suppression, idealization, passive aggression, isolation, autistic fantasy, denial, and dissociation. These results support the previous literature (e.g., Cramer, 2002; Senad, 2022), indicating that men are more likely to rely on defenses involving avoidance and emotional distancing, which may help maintain emotional control and reduce vulnerability.

Regarding personality trait domains, male participants exhibited higher levels of antagonism and disinhibition, suggesting a greater propensity for antisocial and narcissistic tendencies. This is in line with prior research (Granieri et al., 2017; Paris, 2004) linking these traits to externalizing behaviors and difficulties in affect regulation. In contrast, female participants reported higher levels of somatization and negative affectivity, consistent with findings that women are more prone to internalizing disorders (Eaton et al., 2012; Gao et al., 2020; Kramer et al., 2008; Tung et al., 2018).

### 4.4. Limitations and Future Directions

Despite its interesting findings, the current study presents limitations that should be taken into consideration. First, the use of self-report measures may have introduced biases related to introspective accuracy and social desirability. Additionally, the cross-sectional design of the study, the geographically limited sample, and the characteristics of the sample (people from the community) restrict the generalizability of the findings. Moreover, the cross-sectional design precludes any inference about the directionality of the observed associations. For instance, it remains unclear whether certain defense mechanisms precede the development of maladaptive personality traits, or whether these constructs co-develop over time. For these reasons, future research should employ longitudinal designs to deepen the understanding of the complex interactions between attachment styles, defense mechanisms, and personality domains, disentangling the temporal and possibly reciprocal relations between these constructs. Another limitation may concern gender differences: the sociodemographic survey offered only binary response options related to sex (male and female), thus failing to account for non-binary or other gender identities and potentially limiting the representativeness and generalizability of the results. Future research should include larger and more diverse samples. Furthermore, the possible empirical overlap between the constructs assessed may represent an additional limitation. For instance, some defense mechanisms (e.g., acting out) may reflect traits captured by the PID-5 domains (e.g., antagonism and disinhibition). Similarly, for example, the dismissing attachment style may partially align with the AMPD detachment domain. Such intersections could partially explain the strong associations observed between certain defenses and trait domains. Future research should take this issue into account by selecting assessment tools that reduce the risk of conceptual redundancy across the constructs examined. In addition, one domain of the PID-5—antagonism—showed relatively low internal consistency (α = 0.57), which may limit the reliability of the results related to this specific trait. For this reason, researchers might consider using additional instruments for personality assessment. It is also important to acknowledge that additional factors may contribute to a better understanding of the processes under investigation, such as the presence of stressful or traumatic experiences during childhood, emotional regulation capacities, and reflective functioning. Finally, incorporating structured interview measures, such as the Structured Clinical Interview for the DSM-5 Alternative Model for Personality Disorders (SCID-5-AMPD; First et al., 2018), could enhance the reliability of the findings by allowing for a more nuanced assessment of personality functioning.

## 5. Clinical Implications

The findings of this study could have meaningful clinical implications for both assessment and treatment. Maladaptive personality traits, as defined in the AMPD framework, are closely associated with insecure attachment styles and a predominant reliance on neurotic and immature defense mechanisms. This combination of relational insecurity and potentially inadequate defenses may contribute to the development of maladaptive personality functioning, leading to significant distress for the patient and those around them. Our findings suggest the importance of including a thorough clinical assessment of adult attachment and defense mechanisms to better understand how relational history and characteristic ways of managing threats shape the individual’s functioning. Overall, maladaptive personality domains tend to be positively predicted by insecure attachment, and negatively by secure attachment. As such, a key goal in therapy might be the development of what Bowlby (1988) called a “secure base”. Indeed, a sense of safety within the therapeutic relationship provides the foundation for exploring the patient’s internal world, relational patterns, and behaviors. Within this framework, therapy can concurrently foster insight and offer a “corrective emotional experience” (Alexander & French, 1946). Furthermore, the hierarchical regression findings—by identifying predictors of each maladaptive personality domain—may help clinicians to tailor interventions for individuals with elevated scores in specific areas. For instance, for individuals with high levels of negative affectivity, emotional states may be perceived as confusing or excessively intense—lacking clear meaning and thus difficult to tolerate. In such cases, the defense mechanism of idealization may emerge as attempts to cope with this internal confusion via the safety of an external figure, while fantasizing around experiencing different contexts and situations (as suggested by the overuse of autistic fantasy). Within the therapeutic setting, this dynamic can manifest through an idealizing stance toward the therapist, which, if met with empathic containment rather than confrontation, may serve as a transitional scaffold for emotional regulation. Rather than encouraging immediate insight or deeper exploration, clinical work might aim to support the patient in gradually modifying the internal meaning attributed to negative affect, allowing emotional experiences to be held, named, and eventually integrated. This shift—from affect as something to escape to affect as something that can be symbolized—lays the groundwork for a more stable emotional life. In patients with a marked tendency toward detachment—driven by attachment styles characterized by negative representations of others and by the frequent use of projection—it can be challenging to establish a strong therapeutic alliance early in treatment. The clinician, in fact, may become a natural target for projected mistrust, and if these projections are not managed carefully, they can jeopardize the therapeutic relationship, leading to what Safran and Muran (2000) call “withdrawal ruptures”, which require thoughtful repair. For these reasons, it is essential for clinicians to carefully calibrate their level of interpersonal closeness—avoiding both excessive intrusiveness and excessive distance, which may result in collusion or even re-traumatization. Despite the antagonism scale displaying a barely acceptable internal consistency in our sample, in line with the existing literature, the results our findings indicate a frequent reliance on immature defenses, including splitting, denial, and projection. Following Kernberg’s (1975) recommendations, clinical work with these patients might emphasize addressing these primitive defenses and working through the transference, with the goal of helping them to develop greater insight into their personality functioning and its relational consequences. The results concerning the disinhibition domain emphasize the importance of prioritizing efforts to stabilize acting-out behaviors, in order to reduce potential risks to the patient and others. The presence of dissociation also suggests the need for careful assessment of potential trauma and clinically significant dissociative symptoms so that treatment can be tailored accordingly (Schimmenti, 2018; Schimmenti & Caretti, 2016). If acting out occurs alongside dissociative episodes marked by disruptions in consciousness, therapy—once acute symptoms are stabilized—should aim to help the patient recognize and integrate dissociated self-states, ultimately supporting improved emotional and behavioral regulation (Brand, 2024; Chefetz, 2015). These results also underscore the need to prioritize, in patients with high levels of psychoticism, efforts to re-engage with the external world and interpersonal relationships—starting with the therapeutic relationship, through a careful and often lengthy process (Gabbard, 2014). This may represent a significant clinical challenge, requiring the clinician to remain attuned to the therapeutic relationship by containing the patient’s projections and the underlying guilt that might drive the need for undoing. Difficulties in reality testing point to the need for a supportive therapeutic approach, including carefully timed psychoeducational interventions aimed at helping these patients reconnect with external reality and their emotional experience.

## 6. Conclusions

Consistent with the prior literature, the findings of this study suggest that maladaptive personality domains, as conceptualized within the AMPD framework, are generally predicted by the use of immature and neurotic defense mechanisms, as well as by insecure attachment styles. In line with attachment theory, these results highlight the central role of early relational experiences—particularly attachment bonds—in shaping enduring configurations of personality functioning. Bowlby (1988) emphasized the crucial role of a “secure base” in fostering, first in the child and later in the adult, the capacity for the healthy exploration of reality. Attachment insecurity may give rise instead to a need to overuse some defense mechanisms to restore the sense of safety. These mechanisms may serve to protect the individual from threats such as the fear of losing the attachment bonds, loss of control, disruptions in self-coherence, or, in more extreme cases, psychic fragmentation. Over time, adaptations originally deployed to ensure survival in the face of perceived threat may become habitual and increasingly integrated into the personality structure. For this reason, we suggest that in clinical settings, a thorough assessment of personality functioning based on the AMPD model may benefit from also considering individuals’ characteristic ways of relating to others and defending themselves against real or perceived internal and external threats. Its limitations notwithstanding, the present study may thus hold important implications for clinical practice, suggesting the value of integrating personality assessment with the exploration of adult attachment styles and defensive functioning. This approach moves beyond the identification of symptomatology to examine the developmental and relational foundations of psychological distress. Such a perspective underscores the value of an integrated assessment framework aimed at identifying the individual’s characteristic ways of engaging in, or withdrawing from, interpersonal relationships. Furthermore, this approach reinforces the view that psychotherapy should offer a “safe retreat” and a “secure base” from which patients can safely explore their internal world and eventually change their maladaptive ways of functioning.

## Figures and Tables

**Table 1:** Definition of defense mechanisms.

Defense Mechanisms (DSQ-40)	Definition	Example Item
**Mature**		
Anticipation	Mentally preparing for future challenges by experiencing the associated affect in advance.	When I have to face a difficult situation, I try to image what it will be like and plan ways to cope with it.
Humor	Using irony or humor to highlight the absurd aspects of a conflict or distressing experience.	I am usually able to see the funny side of an otherwise painful predicament.
Sublimation	Transforming emotional conflicts into socially acceptable or productive activities.	I work out my anxiety through doing something constructive and creative like painting or woodwork.
Suppression	Consciously choosing not to think about disturbing experiences, impulses, wishes, or emotional conflicts.	I’m able to keep a problem out of my mind until I have time to deal with it.
**Neurotic**		
Idealization	Attributing exaggerated positive qualities to others, often to avoid recognizing their flaws.	I always feel that someone I know is a guardian angel.
Pseudo-altruism	Managing personal conflict or distress by helping others in ways that sustain self-esteem and indirectly express one’s own unmet needs.	I get satisfaction from helping others and if this were taken away from me I would get depressed.
Reaction Formation	Transforming unacceptable impulses or affects into their opposite behaviors or attitudes.	If someone mugged me and stole my money, I’d rather he be helped than punished.
Undoing	Attempting to symbolically reverse or deny unacceptable impulses or behaviors through compensatory actions.	After I fight for my rights, I tend to apologize for my assertiveness.
**Immature**		
Acting Out	Engaging in impulsive behaviors with no regard for consequences to mitigate or avoid awareness of distressing emotional experiences.	I often act impulsively when something is bothering me.
Autistic Fantasy	Replacing interpersonal tension with internal fantasy scenarios, thereby avoiding social contact.	I get more satisfaction from my fantasies than from my real life.
Denial	Refusing or neglecting to acknowledge painful aspects of reality that others can clearly see.	People say I tend to ignore unpleasant facts as if they didn’t exist.
Devaluation	Attributing excessively negative qualities to oneself or others.	I pride myself on my ability to cut people down to size.
Displacement	Redirecting unacceptable feelings or impulses from their original object to a safer substitute.	When I’m depressed or anxious, eating makes me feel better.
Dissociation	Temporarily altering the integrative functions of consciousness or identity to manage internal or external stress.	I ignore danger as if I was Superman.
Isolation	Disconnecting emotional responses from thoughts or situations that would typically elicit them.	I’m often told that I don’t show my feelings.
Passive Aggression	Indirectly expressing hostility or resentment through procrastination, stubbornness, or intentional inefficiency, rather than directly confronting the source of frustration.	If my boss bugged me, I might make a mistake in my work or work more slowly so as to get back at him.
Projection	Attributing one’s own feelings and impulses to others.	People tend to mistreat me.
Rationalization	Justifying one’s or others’ behavior by constructing logical reasons to make it seem acceptable.	I am able to find good reasons for everything I do.
Somatization	Converting psychological distress into physical symptoms, often without a medical explanation, to express internal conflict or obtain care.	I get physically ill when things aren’t going well for me.
Splitting	Rigidly separating positive and negative qualities of the self or others, precluding integration of ambivalent object representations.	As far as I’m concerned, people are either good or bad.

*Note*: All example items are drawn from (Andrews et al., 1993, pp. 254–256).

**Table 2:** Descriptive statistics and sex differences.

	Total Group (N = 400)		Male (N = 190)		Female (N = 210)			
	M	(DS)	M	(DS)	M	(DS)	t(398)	*p*
Age	36.96	(0.5)	37.23	(11.69)	36.72	(11.53)	0.44	0.66
Years of education	16.65	(11.59)	16.24	(3.70)	17.01	(3.32)	−2.21	0.03
Secure attachment style (RQ)	3.30	(3.52)	3.52	(1.67)	3.10	(1.64)	2.55	0.01
Dismissing attachment style (RQ)	2.79	(1.52)	2.87	(1.72)	2.71	(1.50)	0.99	0.32
Preoccupied attachment style (RQ)	2.16	(1.64)	2.39	(1.62)	1.95	(1.40)	2.89	<0.01
Fearful attachment style (RQ)	2.49	(1.66)	2.49	(1.62)	2.49	(1.65)	−0.01	1.00
Sublimation (DSQ-40)	4.28	(1.61)	4.08	(1.97)	4.46	(2.25)	−1.76	0.08
Humor (DSQ-40)	6.16	(2.13)	6.32	(2.25)	6.02	(2.33)	1.32	0.19
Anticipation (DSQ-40)	5.20	(2.29)	5.47	(2.00)	4.96	(2.02)	2.51	0.01
Suppression (DSQ-40)	4.25	(2.02)	4.48	(2.01)	4.05	(1.98)	2.15	0.03
Undoing (DSQ-40)	3.92	(2.01)	3.97	(2.09)	3.88	(2.10)	0.42	0.68
Pseudo-altruism (DSQ-40)	4.03	(2.09)	4.00	(1.94)	4.06	(1.78)	−0.33	0.74
Idealization (DSQ-40)	3.30	(1.85)	3.51	(2.03)	3.11	(2.00)	1.98	0.05
Reaction formation (DSQ-40)	4.35	(2.02)	4.34	(2.03)	4.36	(2.15)	−0.07	0.94
Projection (DSQ-40)	3.12	(2.09)	3.25	(1.81)	3.00	(1.87)	1.38	0.17
Passive aggression (DSQ-40)	3.52	(1.85)	3.79	(2.11)	3.27	(1.90)	2.62	0.01
Acting out (DSQ-40)	4.12	(2.02)	4.20	(2.17)	4.05	(2.20)	0.70	0.49
Isolation (DSQ-40)	3.77	(2.19)	4.22	(2.32)	3.35	(2.25)	3.80	<0.001
Devaluation (DSQ-40)	3.93	(2.32)	4.09	(1.81)	3.78	(1.73)	1.76	0.08
Autistic fantasy (DSQ-40)	3.60	(1.77)	4.07	(2.34)	3.18	(2.27)	3.83	<0.001
Denial (DSQ-40)	2.65	(2.34)	2.95	(1.72)	2.38	(1.54)	3.53	<0.001
Displacement (DSQ-40)	3.14	(1.65)	3.13	(1.73)	3.15	(1.90)	−0.08	0.94
Dissociation (DSQ-40)	3.28	(1.82)	3.53	(1.80)	3.05	(1.65)	2.75	0.01
Splitting (DSQ-40)	3.36	(1.74)	3.52	(2.22)	3.20	(2.15)	1.47	0.14
Rationalization (DSQ-40)	4.84	(2.19)	4.93	(1.85)	4.76	(2.06)	0.89	0.37
Somatization (DSQ-40)	3.83	(1.96)	3.48	(2.11)	4.15	(2.24)	−3.05	<0.01
Negative affectivity (PID-5-BF)	1.15	(2.21)	1.08	(0.64)	1.21	(0.66)	−1.99	0.05
Detachment (PID-5-BF)	0.75	(0.65)	0.81	(0.62)	0.70	(0.60)	1.74	0.08
Antagonism (PID-5-BF)	0.52	(0.61)	0.61	(0.45)	0.43	(0.41)	4.03	<0.001
Disinhibition (PID-5-BF)	0.83	(0.44)	0.91	(0.63)	0.76	(0.52)	2.58	0.01
Psychoticism (PID-5-BF)	0.65	(0.58)	0.70	(0.65)	0.61	(0.63)	1.41	0.16

*Note.* Gender = “male” was coded as 1, “female” was coded as 2, RQ = Relationship Questionnaire, DSQ-40 = Defense Style Questionnaire, PID-5 = The Personality Inventory Brief Form Adult.

**Table 3:** Pearson’s *r* correlation among sociodemographic variables, attachment styles, and maladaptive personality domains.

	2.	3.	4.	5.	6.	7.	8.	9.	10.	11.
1. Age	0.16 **	0.11 *	0.01	−0.24 **	−0.20 **	−0.23 **	−0.07	−0.06	−0.02	−0.16 **
2. Years of education	-	0.11 *	−0.14 **	−0.20 **	−0.09	−0.22 **	−0.22 **	−0.10 *	−0.31 **	−0.27 **
3. Secure attachment style (RQ)		-	−0.18 **	−0.06	−0.13 *	−0.25 **	−0.37 **	−0.10	−0.16 **	−0.24 **
4. Dismissing attachment style (RQ)			-	0.09	0.22 **	0.03	0.28 **	0.14 *	0.16 *	0.13 **
5. Preoccupied attachment style (RQ)				-	0.42 **	0.32 **	0.22 **	0.14 **	0.16 **	0.25 **
6. Fearful attachment style (RQ)					-	−0.24 **	0.37 **	0.11 *	0.11 *	0.19 **
7. Negative affect (PID-5-BF)						-	0.46 **	0.30 **	0.38 **	0.53 **
8. Detachment (PID-5-BF)							-	0.38 **	0.35 **	0.50 **
9. Antagonism (PID-5-BF)								-	0.34 **	0.36 **
10. Disinhibition (PID-5-BF)									-	0.49 **
11. Psychoticism (PID-5-BF)										-

*Note.* RQ = Relationship Questionnaire, PID-5 = The Personality Inventory Brief Form Adult; * *p* < 0.05, ** *p* < 0.01.

**Table 4:** Pearson’s *r* correlation among sociodemographic variables, defense mechanisms, and maladaptive personality domains.

	2.	3.	4.	5.	6.	7.	8.	9.	10.	11.	12.	13.	14.	15.	16.	17.	18.	19.	20.	21.	22.	23.	24.	25.	26.	27.
1. Age	0.16 **	0.02	−0.04	−0.12 *	0.03	−0.04	−0.04	−0.14 **	−0.20 **	−0.08	−0.09	−0.07	−0.14 **	−0.13 **	−0.16 **	0.04	0.01	0.04	−0.07	0.00	−0.07	−0.23 **	−0.07	−0.06	−0.02	−0.16 **
2. Years of education	-	0.06	−0.01	−0.04	−0.06	−0.10 *	−0.04	−0.15 **	0.01	−0.17 **	−0.17 **	−0.20 **	−0.21 **	−0.02	−0.25 **	−0.14 **	−0.03	−0.04	−0.31 **	−0.07	−0.07	−0.22 **	−0.22 **	−0.10 *	−0.31 **	−0.27 **
3. Sublimation (DSQ-40)		-	0.41 **	0.35 **	0.40 **	0.35 **	0.35 **	0.31 **	0.29 **	0.20 **	0.20 **	0.23 **	0.26 **	0.34 **	0.20 **	0.19 **	0.31 **	0.34 **	0.17 **	0.42 **	0.27 **	0.08	−0.04	0.07	0.02	0.10 *
4. Humor (DSQ-40)			-	0.41 **	0.50 **	0.22 **	0.23 **	0.21 **	0.36 **	0.19 **	0.12 *	0.23 **	0.23 **	0.38 **	0.17 **	0.28 **	0.15 **	0.42 **	0.20 **	0.49 **	0.12 *	−0.10 *	−0.08	0.06	0.02	0.05
5. Anticipation (DSQ-40)				-	0.34 **	0.48 **	0.52 **	0.37 **	0.42 **	0.40 **	0.46 **	0.37 **	0.41 **	0.45 **	0.40 **	0.29 **	0.33 **	0.33 **	0.31 **	0.42 **	0.36 **	0.20 **	0.16 *	0.13 *	0.06	0.14 *
6. Suppression (DSQ-40)					-	0.28 **	0.31 **	0.27 **	0.27 **	0.16 **	0.12 *	0.19 **	0.35 **	0.37 **	0.20 **	0.36 **	0.15 **	0.41 **	0.28 **	0.49 **	0.03	−0.09	0.02	0.13 *	0.12 *	0.09
7. Undoing (DSQ-40)						-	0.47 **	0.50 **	0.39 **	0.40 **	0.45 **	0.40 **	0.32 **	0.41 **	0.41 **	0.32 **	0.45 **	0.33 **	0.36 **	0.28 **	0.34 **	0.36 **	0.25 **	0.17	0.26 *	0.31 *
8. Pseudo-altruism (DSQ-40)							-	0.43 **	0.39 **	0.37 **	0.46 **	0.37 **	0.29 **	0.45 **	0.38 **	0.29 **	0.43 **	0.36 **	0.30 **	0.31 **	0.39 **	0.33 **	0.15 **	0.17 *	0.14 *	0.17 *
9. Idealization (DSQ-40)								-	0.37 **	0.41 **	0.42 **	0.42 **	0.29 **	0.34 **	0.36 **	0.36 **	0.34 **	0.33 **	0.45 **	0.27 **	0.31 **	0.39 **	0.18 *	0.19 *	0.27 *	0.33 *
10. Reaction formation (DSQ-40)									-	0.36 **	0.28 **	0.25 **	0.25 **	0.34 **	0.30 **	0.25 **	0.31 **	0.26 **	0.21 **	0.29 **	0.31 **	0.19 **	0.06	0.02	0.04	0.18 *
11. Projection (DSQ-40)										-	0.56 **	0.43 **	0.42 **	0.37 **	0.51 **	0.33 **	0.38 **	0.29 **	0.38 **	0.12 *	0.39 **	0.47 **	0.40 **	0.29 **	0.30 **	0.43 **
12. Passive aggression (DSQ-40)											-	0.50 **	0.43 **	0.41 **	0.53 **	0.28 **	0.45 **	0.26 **	0.43 **	0.19 **	0.48 **	0.43 **	0.34 **	0.28 **	0.33 **	0.32 **
13. Acting out (DSQ-40)												-	0.31 **	0.41 **	0.37 **	0.30 **	0.35 **	0.38 **	0.53 **	0.25 **	0.43 **	0.41 **	0.24 **	0.22 **	0.46 **	0.35 **
14. Isolation (DSQ-40)													-	0.44 **	0.44 **	0.43 **	0.36 **	0.35 **	0.36 **	0.26 **	0.26 **	0.22 **	0.46 **	0.24 **	0.26 **	0.36 **
15. Devaluation (DSQ-40)														-	0.44 **	0.41 **	0.33 **	0.45 **	0.34 **	0.38 **	0.33 **	0.20 **	0.22 **	0.21 **	0.13 **	0.16 **
16. Autistic fantasy (DSQ-40)															-	0.33 **	0.36 **	0.24 **	0.36 **	0.23 **	0.37 **	0.43 **	0.44 **	0.26 **	0.24 **	0.43 **
17. Denial (DSQ-40)																-	0.28 **	0.63 **	0.38 **	0.33 **	0.16 **	0.14 **	0.23 **	0.29 **	0.28 **	0.25 **
18. Displacement (DSQ-40)																	-	0.28 **	0.23 **	0.17 **	0.44 **	0.39 **	0.22 **	0.16 **	0.22 **	0.28 **
19. Dissociation (DSQ-40)																		-	0.30 **	0.52 **	0.17 **	0.07	0.05	0.22 **	0.27 **	0.16 **
20. Splitting (DSQ-40)																			-	0.27 **	0.33 **	0.36 **	0.30 **	0.29 **	0.43 **	0.33 **
21. Rationalization (DSQ-40)																				-	0.15 *	−0.03	−0.06	0.05	0.00	0.02
22. Somatization (DSQ-40)																					-	0.43 **	0.27 **	0.14 **	0.16 **	0.27 **
23. Negative affect (PID-5-BF)																						-	0.46 **	0.30 **	0.38 **	0.53 **
24. Detachment (PID-5-BF)																							-	0.38 **	0.35 **	0.50 **
25. Antagonism (PID-5-BF)																								-	0.34 **	0.36 **
26. Disinhibition (PID-5-BF)																									-	0.49 **
27. Psychoticism (PID-5-BF)																										-

*Note.* DSQ-40 = Defense Style Questionnaire, PID-5 = The Personality Inventory Brief Form Adult; * *p* < 0.05, ** *p* < 0.01.

**Table 5:** Hierarchical regression model predicting the severity of negative affectivity.

	Model 1							Model 2						Model 3				
	B	95% (CI)		β	*t*	*p*	B	95% (CI)		β	*t*	*p*	B	95% (CI)		β	*t*	*p*
Gender	0.15	0.03	0.28	0.12	2.44	0.02	15	0.04	0.27	0.12	2.57	0.01	0.13	0.03	0.23	0.10	2.46	0.01
Age	−0.01	−0.02	−0.01	−0.19	−3.95	<0.001	−0.01	−0.01	0.00	−0.10	−2.06	0.04	−0.01	−0.01	−0.00	−0.12	−3.10	<0.01
Years of education	−0.04	−0.06	−0.02	−0.20	−4.11	<0.001	−0.03	−0.05	−0.01	−0.16	−3.34	<0.01	−0.01	−0.03	0.00	−0.06	−1.47	0.14
Secure attachment style (RQ)							−0.08	−0.11	−0.04	−0.20	−4.34	<0.001	−0.05	−0.08	−0.02	−0.12	−2.97	<0.01
Dismissing attachment style (RQ)							−0.05	−0.09	−0.02	−0.13	−2.74	0.01	−0.04	−0.07	−0.01	−0.09	−2.36	0.02
Preoccupied attachment style (RQ)							0.10	0.06	0.15	0.24	4.78	<0.001	0.03	−0.01	0.07	0.07	1.53	0.13
Fearful attachment style (RQ)							0.04	0.00	0.08	0.11	2.11	0.04	0.01	−0.02	0.04	0.02	0.55	0.59
Sublimation (DSQ-40)													−0.00	−0.03	0.03	−0.01	−0.21	0.83
Humor (DSQ-40)													−0.04	−0.06	−0.01	−0.13	−2.60	0.01
Anticipation (DSQ-40)													−0.02	−0.05	0.01	−0.06	−1.13	0.26
Suppression (DSQ-40)													−0.04	−0.07	−0.01	−0.12	−2.44	0.02
Undoing (DSQ-40)													0.02	−0.01	0.05	0.07	1.43	0.15
Pseudo-altruism (DSQ-40)													0.03	−0.01	0.06	0.08	1.59	0.11
Idealization (DSQ-40)													0.04	0.01	0.07	0.12	2.57	0.01
Reaction formation (DSQ-40)													−0.01	−0.04	0.02	−0.04	−0.77	0.44
Projection (DSQ-40)													0.06	0.03	0.10	0.18	3.57	<0.001
Passive aggression (DSQ-40)													−0.01	−0.04	0.03	−0.02	−0.37	0.71
Acting out (DSQ-40)													0.04	0.01	0.07	0.13	2.72	0.01
Isolation (DSQ-40)													−0.01	−0.04	0.02	−0.04	−0.81	0.42
Devaluation (DSQ-40)													−0.01	−0.04	0.03	−0.02	−0.33	0.74
Autistic fantasy (DSQ-40)													0.04	0.02	0.07	0.15	3.09	<0.01
Denial (DSQ-40)													−0.00	−0.04	0.04	−0.01	−0.21	0.84
Displacement (DSQ-40)													0.06	0.02	0.09	0.16	3.44	<0.01
Dissociation (DSQ-40)													−0.01	−0.05	0.04	−0.01	−0.26	0.80
Splitting (DSQ-40)													0.03	−0.00	0.05	0.09	1.80	0.07
Rationalization (DSQ-40)													−0.02	−0.05	0.01	−0.06	−1.23	0.22
Somatization (DSQ-40)													0.03	−0.00	0.05	0.09	1.88	0.06
*R*^2^ =	0.099						0.224						0.526					
*R*^2^ Change =	0.099						0.126						0.301					
*Model*	F_(3,396)_ = 14.44, *p* < 0.001; B = 1.93							F_(4,392)_ = 16.18, *p* < 0.001; B = 1.97						F_(20,372)_ = 15.27, *p* < 0.001; B = 1.11				

*Note.* Gender = “male” was coded as 1, “female” was coded as 2, RQ = Relationship Questionnaire, DSQ-40 = Defense Style Questionnaire, PID-5-BF = The Personality Inventory Brief Form; Durbin–Watson = 1.90.

**Table 6:** Hierarchical regression model predicting the severity of detachment.

	Model 1							Model 2						Model 3				
	B	95% (CI)		β	*t*	*p*	B	95% (CI)		β	*t*	*p*	B	95% (CI)		β	*t*	*p*
Gender	−0.08	−0.20	0.04	−0.07	−1.32	0.19	−0.12	−0.22	−0.01	−0.10	−2.22	0.03	−0.03	−0.13	0.07	−0.03	−0.62	0.54
Age	−0.00	−0.01	0.00	−0.04	−0.74	0.46	0.00	−0.00	0.01	0.05	1.11	0.27	0.00	−0.00	0.01	0.06	1.35	0.18
Years of education	−0.04	−0.05	−0.02	−0.21	−4.14	<0.001	−0.02	−0.04	−0.01	−0.13	−2.98	<0.01	−0.00	−0.02	0.012	−0.02	−0.35	0.73
Secure attachment style (RQ)							−0.11	−0.15	−0.08	−0.31	−6.97	<0.001	−0.07	−0.10	−0.04	−0.20	−4.90	<0.001
Dismissing attachment style (RQ)							0.05	0.02	0.09	0.14	3.04	<0.01	0.05	0.02	0.08	0.13	3.03	<0.01
Preoccupied attachment style (RQ)							0.02	−0.02	0.06	0.05	0.95	0.35	−0.01	−0.05	0.02	−0.03	−0.69	0.49
Fearful attachment style (RQ)							0.10	0.07	0.14	0.28	5.73	<0.001	0.06	0.03	0.10	0.16	3.68	<0.001
Sublimation (DSQ-40)													−0.04	−0.07	−0.01	−0.14	−3.02	<0.01
Humor (DSQ-40)													−0.02	−0.05	0.01	−0.08	−1.63	0.10
Anticipation (DSQ-40)													0.000	−0.03	0.03	0.00	−0.00	0.10
Suppression (DSQ-40)													−0.01	−0.04	0.03	−0.02	−0.32	0.75
Undoing (DSQ-40)													0.03	−0.00	0.06	0.10	1.89	0.06
Pseudo-altruism (DSQ-40)													0.00	−0.03	0.04	0.01	0.22	0.83
Idealization (DSQ-40)													0.00	−0.03	0.03	0.00	0.04	0.10
Reaction formation (DSQ-40)													−0.02	−0.05	0.01	−0.08	−1.61	0.11
Projection (DSQ-40)													0.03	0.00	0.07	0.10	1.95	0.05
Passive aggression (DSQ-40)													−0.01	−0.05	0.02	−0.05	−0.86	0.39
Acting out (DSQ-40)													0.01	−0.02	0.04	0.03	0.54	0.59
Isolation (DSQ-40)													0.07	0.04	0.10	0.26	5.11	<0.001
Devaluation (DSQ-40)													0.01	−0.03	0.04	0.01	0.27	0.79
Autistic fantasy (DSQ-40)													0.05	0.02	0.07	0.18	3.53	<0.001
Denial (DSQ-40)													0.03	−0.01	0.07	0.07	1.36	0.18
Displacement (DSQ-40)													−0.01	−0.04	0.03	−0.02	−0.39	0.69
Dissociation (DSQ-40)													−0.04	−0.07	0.00	−0.10	−1.76	0.08
Splitting (DSQ-40)													0.02	−0.00	0.05	0.08	1.66	0.10
Rationalization (DSQ-40)													−0.02	−0.05	0.01	−0.06	−1.23	0.22
Somatization (DSQ-40)													0.03	−0.00	0.05	0.09	1.88	0.06
*R*^2^ =	0.054						0.295						0.489					
*R*^2^ Change =	0.054						0.241						0.194					
*Model*	F_(3,396)_ = 7.47, *p* < 0.001; B = 1.55							F_(4,392)_ = 23.39, *p* < 0.001; B = 1.15						F_(20,372)_ = 13.17, *p* = 0.02; B = 0.47				

*Note.* Gender = “male” was coded as 1, “female” was coded as 2, RQ = Relationship Questionnaire, DSQ-40 = Defense Style Questionnaire, PID-5-BF = The Personality Inventory Brief Form; Durbin–Watson = 1.90.

**Table 7:** Hierarchical regression model predicting the severity of antagonism.

	Model 1							Model 2						Model 3				
	B	95% (CI)		β	*t*	*p*	B	95% (CI)		β	*t*	*p*	B	95% (CI)		β	*t*	*p*
Gender	−0.17	−0.25	−0.08	−0.19	−3.88	<0.001	−0.17	−0.25	−0.08	−0.19	−3.83	<0.001	−0.13	−0.22	−0.04	−0.15	−2.83	0.01
Age	−0.00	−0.00	0.00	−0.05	−1.09	0.28	−0.00	−0.01	0.00	−0.03	−0.50	0.62	−0.00	−0.01	0.00	−0.08	−1.62	0.11
Years of education	−0.01	−0.02	0.00	−0.07	−1.39	0.16	−0.00	−0.02	0.01	−0.04	−0.69	0.49	0.01	−0.01	0.02	0.06	1.19	0.23
Secure attachment style (RQ)							−0.02	−0.05	0.00	−0.09	−1.76	0.08	−0.01	−0.03	0.02	−0.03	−0.60	0.55
Dismissing attachment style (RQ)							0.02	−0.00	0.05	0.09	1.75	0.08	0.02	−0.01	0.05	0.08	1.54	0.13
Preoccupied attachment style (RQ)							0.02	−0.01	0.05	0.07	1.29	0.20	0.01	−0.03	0.04	0.03	0.42	0.67
Fearful attachment style (RQ)							0.01	−0.02	0.04	0.04	0.73	0.47	−0.00	−0.03	0.03	−0.02	−0.28	0.78
Sublimation (DSQ-40)													0.00	−0.02	0.03	0.02	0.34	0.73
Humor (DSQ-40)													−0.00	0.02	0.02	−0.01	−0.08	0.94
Anticipation (DSQ-40)													−0.01	−0.04	0.01	−0.06	−0.96	0.34
Suppression (DSQ-40)													0.01	−0.02	0.04	0.05	0.81	0.42
Undoing (DSQ-40)													0.00	−0.02	0.03	0.01	0.08	0.93
Pseudo-altruism (DSQ-40)													0.01	−0.02	0.04	0.05	0.86	0.39
Idealization (DSQ-40)													−0.01	−0.03	0.02	−0.02	−0.34	0.73
Reaction formation (DSQ-40)													−0.03	−0.06	−0.01	−0.16	−2.78	0.01
Projection (DSQ-40)													0.03	0.00	0.06	0.14	2.25	0.03
Passive aggression (DSQ-40)													0.02	−0.01	0.05	0.09	1.31	0.19
Acting out (DSQ-40)													−0.00	−0.03	0.02	−0.02	−0.29	0.77
Isolation (DSQ-40)													−0.00	−0.03	0.02	−0.02	−0.33	0.74
Devaluation (DSQ-40)													0.00	−0.03	0.03	−0.00	−0.03	0.98
Autistic fantasy (DSQ-40)													0.01	−0.01	0.03	0.05	0.78	0.44
Denial (DSQ-40)													0.05	0.00	0.07	0.13	2.02	0.04
Displacement (DSQ-40)													0.00	−0.03	0.03	0.00	0.05	0.96
Dissociation (DSQ-40)													0.02	−0.02	0.05	0.07	0.96	0.34
Splitting (DSQ-40)													0.03	0.01	0.06	0.17	2.76	0.01
Rationalization (DSQ-40)													−0.02	−0.05	0.01	−0.09	−1.42	0.16
Somatization (DSQ-40)													−0.13	−0.02	0.03	0.02	0.33	0.74
*R*^2^ =	0.048						0.078						0.219					
*R*^2^ Change =	0.048						0.030						0.141					
*Model*	F_(3,396)_ = 6.68, *p* < 0.001; B = 0.99							F_(4,392)_ = 4.74, *p* < 0.001; B = 0.82						F_(20,372)_ = 3.87, *p* = 0.02; B = 0.42				

*Note.* Gender = “male” was coded as 1, “female” was coded as 2, RQ = Relationship Questionnaire, DSQ-40 = Defense Style Questionnaire, PID-5-BF = The Personality Inventory Brief Form; Durbin-Watson = 1.92.

**Table 8:** Hierarchical regression model predicting the severity of disinhibition.

	Model 1							Model 2						Model 3				
	B	95% (CI)		β	*t*	*p*	B	95% (CI)		β	*t*	*p*	B	95% (CI)		β	*t*	*p*
Gender	−0.11	−0.22	0.00	−0.09	−1.96	0.05	−0.12	−0.23	−0.01	−0.10	−2.07	0.04	−0.08	−0.18	0.03	−0.07	−1.44	0.15
Age	0.00	−0.00	0.01	0.02	0.49	0.63	0.00	−0.00	0.01	0.06	1.14	0.26	0.00	−0.00	0.00	−0.00	−0.04	0.97
Years of education	−0.05	−0.07	−0.04	−0.31	−6.31	<0.001	−0.04	−0.06	−0.03	−0.27	−5.50	<0.001	−0.02	−0.04	−0.01	−0.13	−2.95	<0.01
Secure attachment style (RQ)							−0.05	−0.08	−0.01	−0.13	−2.67	0.01	−0.03	−0.06	−0.00	−0.10	−2.24	0.03
Dismissing attachment style (RQ)							0.03	−0.01	0.06	0.08	1.53	0.13	0.03	−0.01	0.06	0.07	1.60	0.11
Preoccupied attachment style (RQ)							0.03	−0.01	0.07	0.07	1.35	0.18	0.00	−0.03	0.04	0.01	0.17	0.87
Fearful attachment style (RQ)							0.01	−0.02	0.05	0.04	0.72	0.47	0.00	−0.03	0.03	0.00	0.07	0.94
Sublimation (DSQ-40)													−0.01	−0.04	0.01	−0.05	−0.94	0.35
Humor (DSQ-40)													−0.01	−0.03	0.02	−0.03	−0.59	0.56
Anticipation (DSQ-40)													−0.04	−0.07	−0.01	−0.15	−2.54	0.01
Suppression (DSQ-40)													0.02	−0.01	0.05	0.08	1.47	0.14
Undoing (DSQ-40)													0.03	−0.00	0.06	0.10	1.77	0.08
Pseudo-altruism (DSQ-40)													−0.01	−0.04	0.03	−0.02	−0.35	0.73
Idealization (DSQ-40)													0.00	−0.03	0.03	0.01	0.24	0.81
Reaction formation (DSQ-40)													−0.02	−0.04	0.01	−0.06	−1.10	0.27
Projection (DSQ-40)													0.01	−0.02	0.04	0.03	0.56	0.58
Passive aggression (DSQ-40)													0.03	−0.01	0.06	0.09	1.55	0.12
Acting out (DSQ-40)													0.08	0.05	0.11	0.30	5.50	<0.001
Isolation (DSQ-40)													0.01	−0.02	0.03	0.02	0.44	0.66
Devaluation (DSQ-40)													−0.04	−0.08	−0.01	−0.12	−2.24	0.03
Autistic fantasy (DSQ-40)													−0.00	−0.03	0.02	−0.02	−0.28	0.78
Denial (DSQ-40)													0.01	−0.03	0.05	0.02	0.28	0.78
Displacement (DSQ-40)													0.02	−0.01	0.05	0.07	1.35	0.18
Dissociation (DSQ-40)													0.07	0.03	0.11	0.20	3.30	<0.01
Splitting (DSQ-40)													0.05	0.02	0.08	0.20	3.64	<0.001
Rationalization (DSQ-40)													−0.05	−0.08	−0.02	−0.17	−3.08	<0.01
Somatization (DSQ-40)													−0.01	−0.04	0.01	−0.05	−1.03	0.30
*R*^2^ =	0.107						0.144						0.414					
*R*^2^ Change =	0.107						0.037						0.270					
*Model*	F_(3,396)_ = 15.81, *p* < 0.001; B = 1.79							F_(4,392)_ = 9.39, *p* < 0.001; B = 1.62						F_(20,372)_ = 9.74, *p* < 0.001; B = 1.06				

*Note.* Gender = “male” was coded as 1, “female” was coded as 2, RQ = Relationship Questionnaire, DSQ-40 = Defense Style Questionnaire, PID-5-BF = The Personality Inventory Brief Form; Durbin–Watson = 2.23.

**Table 9:** Hierarchical regression model predicting the severity of psychoticism.

	Model 1							Model 2						Model 3				
	B	95% (CI)		β	*t*	*p*	B	95% (CI)		β	*t*	*p*	B	95% (CI)		β	*t*	*p*
Gender	−0.06	−0.18	0.06	−0.05	−0.94	0.35	−0.07	−0.19	0.05	−0.05	−1.09	0.28	−0.02	−0.14	0.10	−0.07	−0.36	0.72
Age	−0.01	−0.01	−0.00	−0.12	−2.51	0.01	−0.00	−0.01	0.00	−0.06	−1.27	0.21	−0.00	−0.01	0.00	−0.08	−1.73	0.09
Years of education	−0.05	−0.06	−0.03	−0.25	−5.11	<0.001	−0.04	−0.05	−0.02	−0.20	−4.14	<0.001	−0.02	−0.03	0.00	−0.08	−1.73	0.09
Secure attachment style (RQ)							−0.08	−0.11	−0.04	−0.19	−4.05	<0.001	−0.05	−0.09	−0.08	−0.13	−2.94	<0.01
Dismissing attachment style (RQ)							0.01	−0.02	0.05	0.03	0.64	0.52	−0.00	−0.04	0.03	−0.00	−0.08	0.93
Preoccupied attachment style (RQ)							0.06	0.02	0.11	0.15	2.78	0.01	0.02	−0.03	0.06	0.04	0.70	0.48
Fearful attachment style (RQ)							0.03	−0.02	0.07	0.06	1.21	0.23	−0.01	−0.05	0.02	−0.04	−0.72	0.47
Sublimation (DSQ-40)													0.00	−0.03	0.03	0.00	0.09	0.93
Humor (DSQ-40)													0.00	−0.03	0.03	0.01	0.21	0.83
Anticipation (DSQ-40)													−0.05	−0.08	−0.01	−0.15	−2.48	0.01
Suppression (DSQ-40)													0.00	−0.03	0.04	0.01	0.11	0.92
Undoing (DSQ-40)													0.04	0.00	0.07	0.12	2.16	0.03
Pseudo-altruism (DSQ-40)													−0.02	−0.06	0.02	−0.05	−0.93	0.35
Idealization (DSQ-40)													0.03	−0.01	0.06	0.08	1.53	0.13
Reaction formation (DSQ-40)													0.00	−0.03	0.03	0.01	0.15	0.88
Projection (DSQ-40)													0.06	0.02	0.10	0.17	3.00	<0.01
Passive aggression (DSQ-40)													−0.03	−0.07	0.01	−0.10	−1.60	0.11
Acting out (DSQ-40)													0.04	0.01	0.07	0.14	2.45	0.02
Isolation (DSQ-40)													0.04	0.01	0.07	0.16	2.84	0.01
Devaluation (DSQ-40)													−0.05	−0.09	−0.01	−0.15	−2.65	0.01
Autistic fantasy (DSQ-40)													0.06	0.03	0.09	0.27	4.01	<0.001
Denial (DSQ-40)													0.10	−0.04	0.05	0.02	0.39	0.70
Displacement (DSQ-40)													0.02	−0.01	0.06	0.06	1.19	0.24
Dissociation (DSQ-40)													0.02	−0.03	0.06	0.04	0.66	0.51
Splitting (DSQ-40)													0.02	−0.02	0.05	0.05	0.99	0.33
Rationalization (DSQ-40)													−0.03	−0.07	0.00	−0.10	−1.78	0.08
Somatization (DSQ-40)													0.02	−0.02	0.05	0.05	0.96	0.34
*R*^2^ =	0.091						0.166						0.388					
*R*^2^ Change =	0.091						0.075						0.221					
*Model*	F_(3,396)_ = 13.28, *p* < 0.001; B = 1.75							F_(4,392)_ = 11.16, *p* < 0.001; B = 1.50						F_(20,372)_ = 8.72, *p* = 0.001; B = 0.78				

*Note.* Gender = “male” was coded as 1, “female” was coded as 2, RQ = Relationship Questionnaire, DSQ-40 = Defense Style Questionnaire, PID-5-BF = The Personality Inventory Brief Form; Durbin–Watson = 1.92.

## Data Availability

Data will be available upon reasonable request from the corresponding author (J.C.).

## References

[B1-behavsci-15-01154] Aaronson C. J., Bender D. S., Skodol A. E., Gunderson J. G. (2006). Comparison of attachment styles in borderline personality disorder and obsessive-compulsive personality disorder. Psychiatric Quarterly.

[B2-behavsci-15-01154] Ainsworth M. D. S., Blehar M. C., Waters E., Wall S. (1978). Patterns of attachment: A psychological study of the strange situation.

[B3-behavsci-15-01154] Al-Dajani N., Gralnick T. M., Bagby R. M. (2016). A psychometric review of the personality inventory for DSM–5 (PID–5): Current status and future directions. Journal of Personality Assessment.

[B4-behavsci-15-01154] Alexander F., French T. M. (1946). Psychoanalytic therapy: Principles and application.

[B5-behavsci-15-01154] American Psychiatric Association (2013). Diagnostic and statistical manual of mental disorders.

[B6-behavsci-15-01154] American Psychiatric Association (2022). Diagnostic and statistical manual of mental disorders.

[B7-behavsci-15-01154] American Psychiatric Association [APA] (2000). Diagnostic and statistical manual of mental disorders.

[B8-behavsci-15-01154] American Psychological Association (2018). APA dictionary of psychology.

[B9-behavsci-15-01154] Andrews G., Pollock C., Stewart G. (1989). The determination of defense style by questionnaire. Archives of General Psychiatry.

[B10-behavsci-15-01154] Andrews G., Singh M., Bond M. (1993). Defense style questionnaire-40 (DSQ-40) *[Database record]*.

[B11-behavsci-15-01154] Anzani A., Di Sarno M., Sacchi S., Prunas A. (2018). Maladaptive personality traits, defense mechanisms, and trans-negative attitudes. International Journal of Transgenderism.

[B12-behavsci-15-01154] Ball Cooper E., Anderson J. L., Sharp C., Langley H. A., Venta A. (2021). Attachment, mentalization, and criterion B of the alternative DSM-5 model for personality disorders (AMPD). Borderline Personality Disorder and Emotion Dysregulation.

[B13-behavsci-15-01154] Bartholomew K., Horowitz L. M. (1991). Attachment styles among young adults: A test of a four-category model. Journal of Personality and Social Psychology.

[B14-behavsci-15-01154] Beeney J. E., Wright A. G. C., Stepp S. D., Hallquist M. N., Lazarus S. A., Beeney J. R. S., Scott L. N., Pilkonis P. A. (2017). Disorganized attachment and personality functioning in adults: A latent class analysis. Personality Disorders.

[B15-behavsci-15-01154] Benítez Camacho E., Chávez-León E., Ontiveros Uribe M. P., Yunes Jiménez A., Náfate Lòpez O. (2010). The levels of psychological functioning of personality and the mechanisms of defense. Salud Mental.

[B16-behavsci-15-01154] Bennett C. S. (2005). Attachment theory and research applied to the conceptualization and treatment of pathological narcissism. Clinical Social Work Journal.

[B17-behavsci-15-01154] Botbol M. (2010). Towards an integrative neuroscientific and psychodynamic approach to the transmission of attachment. Journal of Physiology-Paris.

[B18-behavsci-15-01154] Bowins B. (2004). Psychological defense mechanisms: A new perspective. The American Journal of Psychoanalysis.

[B19-behavsci-15-01154] Bowlby J. (1969). Attachment and loss. Vol. I: Attachment.

[B20-behavsci-15-01154] Bowlby J. (1973). Attachment and loss. Vol. 2: Separation: Anxiety and anger.

[B21-behavsci-15-01154] Bowlby J. (1980). Attachment and loss. Vol. 3: Loss, sadness and depression.

[B22-behavsci-15-01154] Bowlby J. (1988). A secure base: Parent-child attachment and healthy human development.

[B23-behavsci-15-01154] Brand B. L. (2024). The concise guide to the assessment and treatment of trauma-related dissociation.

[B24-behavsci-15-01154] Brennan K. A., Shaver P. R. (1998). Attachment styles and personality disorders: Their connections to each other and to parental divorce, parental death, and perceptions of parental caregiving. Journal of Personality.

[B25-behavsci-15-01154] Buchheim A., Diamond D. (2018). Attachment and borderline personality disorder. The Psychiatric Clinics of North America.

[B26-behavsci-15-01154] Caligor E., Levy K. N., Yeomans F. E. (2015). Narcissistic personality disorder: Diagnostic and clinical challenges. The American Journal of Psychiatry.

[B27-behavsci-15-01154] Campbell W. K., Bush C. P., Brunell A. B., Shelton J. (2005). Understanding the social costs of narcissism: The case of the tragedy of the commons. Personality & Social Psychology Bulletin.

[B28-behavsci-15-01154] Carli L. (1995). Attaccamento e rapporto di coppia: Il modello di Bowlby nell’interpretazione del ciclo di vita.

[B29-behavsci-15-01154] Carnelley K. B., Bejinaru M. M., Otway L., Baldwin D. S., Rowe A. C. (2018). Effects of repeated attachment security priming in outpatients with primary depressive disorders. Journal of Affective Disorders.

[B30-behavsci-15-01154] Charles S. T., Reynolds C. A., Gatz M. (2001). Age-related differences and change in positive and negative affect over 23 years. Journal of Personality and Social Psychology.

[B31-behavsci-15-01154] Chefetz R. A. (2015). Intensive psychotherapy for persistent dissociative processes: The fear of feeling real.

[B32-behavsci-15-01154] Clark L. A., Watson D., John O. P., Robins R. W., Pervin L. A. (2008). Temperament: An organizing paradigm for trait psychology. Handbook of personality: Theory and research.

[B33-behavsci-15-01154] Connors M. E. (1997). The renunciation of love: Dismissive attachment and its treatment. Psychoanalytic Psychology.

[B34-behavsci-15-01154] Cooke J. E., Kochendorfer L. B., Stuart-Parrigon K. L., Koehn A. J., Kerns K. A. (2019). Parent-child attachment and children’s experience and regulation of emotion: A meta-analytic review. Emotion.

[B35-behavsci-15-01154] Corruble E., Bronnec M., Falissard B., Hardy P. (2004). Defense styles in depressed suicide attempters. Psychiatry and Clinical Neurosciences.

[B36-behavsci-15-01154] Cramer P. (1991). The development of defense mechanisms: Theory, research, and assessment.

[B37-behavsci-15-01154] Cramer P., Bornstein R. F., Masling J. M. (2002). The study of defense mechanisms: Gender implications. The psychodynamics of gender and gender role.

[B38-behavsci-15-01154] Cramer P. (2006). Protecting the self: Defense mechanisms in action.

[B39-behavsci-15-01154] Cramer P. (2015). Understanding defense mechanisms. Psychodynamic Psychiatry.

[B40-behavsci-15-01154] Cushman F. (2020). Rationalization is rational. Behavioral and Brain Sciences.

[B41-behavsci-15-01154] Del Giudice M. (2011). Sex differences in romantic attachment: A meta-analysis. Personality and Social Psychology Bulletin.

[B42-behavsci-15-01154] Di Giuseppe M., Perry J. C., Conversano C., Gelo O. C. G., Gennaro A. (2020a). Defense mechanisms, gender, and adaptiveness in emerging personality disorders in adolescent outpatients. The Journal of Nervous and Mental Disease.

[B43-behavsci-15-01154] Di Giuseppe M., Perry J. C., Lucchesi M., Michelini M., Vitiello S., Piantanida A., Fabiani M., Maffei S., Conversano C. (2020b). Preliminary reliability and validity of the DMRS-SR-30, a novel self-report measure based on the Defense Mechanisms Rating Scales. Frontiers in Psychiatry.

[B44-behavsci-15-01154] Di Pierro R., Benzi I. M. A., Madeddu F. (2015). Difficulties in emotion regulation among inpatients with substance use disorders: The mediating effect of mature defenses mechanisms. Clinical Neuropsychiatry: Journal of Treatment Evaluation.

[B45-behavsci-15-01154] Eaton N. R., Keyes K. M., Krueger R. F., Balsis S., Skodol A. E., Markon K. E., Grant B. F., Hasin D. S. (2012). An invariant dimensional liability model of gender differences in mental disorder prevalence: Evidence from a national sample. Journal of Abnormal Psychology.

[B46-behavsci-15-01154] Erkoreka L., Zamalloa I., Rodriguez S., Muñoz P., Mendizabal I., Zamalloa M. I., Arrue A., Zumarraga M., Gonzalez-Torres M. A. (2022). Attachment anxiety as mediator of the relationship between childhood trauma and personality dysfunction in borderline personality disorder. Clinical Psychology & Psychotherapy.

[B47-behavsci-15-01154] Evren C., Cınar O., Evren B., Ulku M., Karabulut V., Umut G. (2013). The mediator roles of trait anxiety, hostility, and impulsivity in the association between childhood trauma and dissociation in male substance-dependent inpatients. Comprehensive Psychiatry.

[B48-behavsci-15-01154] Farma T., Cortinovis I. (2000). Misurare i meccanismi di diffesa attraverso il “Defense Style Questionnaire” a 40 item. Attendibilita’ dello strumento e suo utilizzo nel contesto Italiano [Measuring defense mechanism through the 40 items of the “Defense Style Questionnaire.” Reliability of the instrument and its use in the Italian context]. Ricerche di Psicologia.

[B49-behavsci-15-01154] First M. B., Skodol A. E., Bender D. S., Oldham J. M. (2018). Structured clinical interview for the DSM-5 alternative model for personality disorders (SCID-5-AMPD).

[B50-behavsci-15-01154] Fonagy P., Clarkin J. F., Fonagy P., Gabbard G. O. (2010). Attachment and personality pathology. Psychodynamic psychotherapy for personality disorders: A clinical handbook.

[B51-behavsci-15-01154] Fonagy P., Gergely G., Jurist E. L., Target M. (2002). Affect regulation, mentalization, and the development of the self.

[B52-behavsci-15-01154] Fonagy P., Target M. (2005). Bridging the transmission gap: An end to an important mystery of attachment research?. Attachment & Human Development.

[B53-behavsci-15-01154] Fossati A., Krueger R. F., Markon K. E., Borroni S., Maffei C. (2013). Reliability and validity of the personality inventory for DSM-5 (PID-5): Predicting DSM-IV personality disorders and psychopathy in community-dwelling Italian adults. Assessment.

[B54-behavsci-15-01154] Fossati A., Somma A., Borroni S., Markon K. E., Krueger R. F. (2015). The personality inventory for DSM-5 brief form: Evidence for reliability and construct validity in a sample of community-dwelling Italian adolescents. Assessment.

[B55-behavsci-15-01154] Freud A. (1937). The ego and the mechanisms of defense.

[B56-behavsci-15-01154] Frosch J. (1977). The relation between acting out and disorders of impulse control. Psychiatry.

[B57-behavsci-15-01154] Gabbard G. O. (2014). Psychodynamic psychiatry in clinical practice.

[B58-behavsci-15-01154] Gao W., Ping S., Liu X. (2020). Gender differences in depression, anxiety, and stress among college students: A longitudinal study from China. Journal of Affective Disorders.

[B59-behavsci-15-01154] Garza-Guerrero C. (2000). Idealization and mourning in love relationships: Normal and pathological spectra. The Psychoanalytic Quarterly.

[B60-behavsci-15-01154] Gervasi A. M., La Marca L., Lombardo E., Mannino G., Iacolino C., Schimmenti A. (2017). Maladaptive personality traits and internet addiction symptoms among young adults: A study based on the alternative DSM-5 model for personality disorders. Clinical Neuropsychiatry.

[B61-behavsci-15-01154] Gioia F., Imperato C., Boursier V., Franceschini C., Schimmenti A., Musetti A. (2023). The role of defense styles and psychopathological symptoms on adherence to conspiracy theories during the COVID-19 pandemic. Scientific Reports.

[B62-behavsci-15-01154] Gori A., Craparo G., Caretti V., Giannini M., Iraci-Sareri G., Bruschi A., Janiri L., Ponti L., Tani F. (2016). Impulsivity, alexithymia and dissociation among pathological gamblers in different therapeutic settings: A multisample comparison study. Psychiatry Research.

[B63-behavsci-15-01154] Granieri A., La Marca L., Mannino G., Giunta S., Guglielmucci F., Schimmenti A. (2017). The relationship between defense patterns and DSM-5 maladaptive personality domains. Frontiers in Psychology.

[B64-behavsci-15-01154] Gratz K. L. (2006). Risk factors for deliberate self-harm among female college students: The role and interaction of childhood maltreatment, emotional inexpressivity, and affect intensity/reactivity. The American Journal of Orthopsychiatry.

[B65-behavsci-15-01154] Gratz K. L., Tull M. T., Reynolds E. K., Bagge C. L., Lejuez C. W. (2009). Extending extant models of the pathogenesis of borderline personality disorder to childhood borderline personality symptoms: The roles of affective dysfunction, disinhibition, and self-and emotion-regulation deficits. Development and Psychopathology.

[B66-behavsci-15-01154] Griffin D. W., Bartholomew K. (1994). Models of the self and other: Fundamental dimensions underlying measures of adult attachment. Journal of Personality and Social Psychology.

[B67-behavsci-15-01154] Grijalva E., Newman D. A., Tay L., Donnellan M. B., Harms P. D., Robins R. W., Yan T. (2015). Gender differences in narcissism: A meta-analytic review. Psychological Bulletin.

[B68-behavsci-15-01154] Hibbard S., Porcerelli J. (1998). Further validation of the cramer defense mechanism manual. Journal of Personality Assessment.

[B69-behavsci-15-01154] Kernberg O. F. (1975). Borderline conditions and pathological narcissism.

[B70-behavsci-15-01154] Kernberg O. F., Ronningstam E. F. (1998). Pathological narcissism and narcissistic personality disorder: Theoretical background and diagnostic classification. Disorders of narcissism: Diagnostic, clinical, and empirical implications.

[B71-behavsci-15-01154] Klein M. (1946). Notes on some schizoid mechanisms. The International Journal of Psychoanalysis.

[B72-behavsci-15-01154] Kramer M. D., Krueger R. F., Hicks B. M. (2008). The role of internalizing and externalizing liability factors in accounting for gender differences in the prevalence of common psychopathological syndromes. Psychological Medicine.

[B73-behavsci-15-01154] Krueger R. F., Derringer J., Markon K. E., Watson D., Skodol A. E. (2012a). Initial construction of a maladaptive personality trait model and inventory for DSM-5. Psychological Medicine.

[B74-behavsci-15-01154] Krueger R. F., Derringer J., Markon K. E., Watson D., Skodol A. E. (2012b). Personality inventory for DSM-5 (PID-5) *[Database record]*.

[B75-behavsci-15-01154] Lachowicz-Tabaczek K., Lewandowska B., Kochan-Wójcik M., Andrzejewska B. E., Juszkiewicz A. (2021). Grandiose and vulnerable narcissism as predictors of the tendency to objectify other people. Current Psychology.

[B76-behavsci-15-01154] Levy K. N., Johnson B. N., Clouthier T. L., Scala J. W., Temes C. M. (2015). An attachment theoretical framework for personality disorders. Canadian Psychology/Psychologie Canadienne.

[B77-behavsci-15-01154] Lingiardi V., McWilliams N. (2017). Psychodynamic diagnostic manual.

[B78-behavsci-15-01154] Lingiardi V., McWilliams N., Bornstein R. F., Gazzillo F., Gordon R. M. (2015). The psychodynamic diagnostic manual version 2 (PDM-2): Assessing patients for improved clinical practice and research. Psychoanalytic Psychology.

[B79-behavsci-15-01154] Little R. (2020). Engaging with the schizoid compromise: A response to Erskine’s “relational withdrawal, attunement to silence: Psychotherapy of the schizoid process”. International Journal of Integrative Psychotherapy.

[B80-behavsci-15-01154] Lorenzini N., Fonagy P. (2013). Attachment and personality disorders: A short review. Focus.

[B81-behavsci-15-01154] Luyten P., Fonagy P., Holmes P., Farnfield S. (2014). Mentalising in attachment contexts. The Routledge handbook of attachment: Theory.

[B82-behavsci-15-01154] Lynam D. R., Miller J. D. (2019). The basic trait of Antagonism: An unfortunately underappreciated construct. Journal of Research in Personality.

[B83-behavsci-15-01154] Main M., Hesse E., Greenberg M. T., Cicchetti D., Cummings E. M. (1990). Parents’ unresolved traumatic experiences are related to infant disorganized attachment status: Is frightened and/or frightening parental behavior the linking mechanism?. Attachment in the preschool years: Theory, research, and intervention.

[B84-behavsci-15-01154] Main M., Solomon J., Greenberg M. T., Cicchetti D., Cummings E. M. (1990). Procedures for identifying infants as disorganized/disoriented during the Ainsworth Strange Situation. Attachment in the preschool years: Theory, research, and intervention.

[B85-behavsci-15-01154] McGuire A., Gillath O., Jackson Y., Ingram R. (2018). Attachment security priming as a potential intervention for depressive symptoms. Journal of Social and Clinical Psychology.

[B86-behavsci-15-01154] McWilliams N. (2006). Some thoughts about schizoid dynamics. Psychoanalytic Review.

[B87-behavsci-15-01154] McWilliams N. (2011). Psychoanalytic diagnosis: Understanding personality structure in the clinical process.

[B88-behavsci-15-01154] Midolo L. R., Santoro G., Ferrante E., Pellegriti P., Russo S., Costanzo A., Schimmenti A. (2020). Childhood trauma, attachment and psychopathology: A correlation network approach. Mediterranean *Journal of Clinical Psychology*.

[B89-behavsci-15-01154] Mikulincer M., Shaver P. R. (2016). Attachment in adulthood: Structure, dynamics, and change.

[B90-behavsci-15-01154] Miller J. D., Lynam D. R. (2019). The handbook of antagonism: Conceptualizations, assessment, consequences, and treatment of the low end of agreeableness.

[B91-behavsci-15-01154] Mostajabi J., Wright A. G. C. (2024). An exploratory study on disinhibition and interpersonal outcomes in daily life. Personality Disorders: Theory, Research, and Treatment.

[B92-behavsci-15-01154] Mroczek D. K., Kolarz C. M. (1998). The effect of age on positive and negative affect: A developmental perspective on happiness. Journal of Personality and Social Psychology.

[B93-behavsci-15-01154] Mulder R. T. (2024). Personality disorders. The British Journal of Psychiatry.

[B94-behavsci-15-01154] Mullins-Sweatt S. N., DeShong H. L., Lengel G. J., Helle A. C., Krueger R. F. (2019). Disinhibition as a unifying construct in understanding how personality dispositions undergird psychopathology. Journal of Research in Personality.

[B95-behavsci-15-01154] Muris P., Winands D., Horselenberg R. (2003). Defense styles, personality traits, and psychopathological symptoms in nonclinical adolescents. Journal of Nervous and Mental Disease.

[B96-behavsci-15-01154] Musslick S., Cohen J. D. (2021). Rationalizing constraints on the capacity for cognitive control. Trends in Cognitive Sciences.

[B97-behavsci-15-01154] Nigg J. T., Silk K. R., Stavro G., Miller T. (2005). Disinhibition and borderline personality disorder. Development and Psychopathology.

[B98-behavsci-15-01154] Nolte T., Guiney J., Fonagy P., Mayes L. C., Luyten P. (2011). Interpersonal stress regulation and the development of anxiety disorders: An attachment-based developmental framework. Frontiers in Behavioral Neuroscience.

[B99-behavsci-15-01154] Nunes F., Mota C. P., Ferreira T., Schoon I., Matos P. M. (2022). Parental meta-emotion, attachment to parents, and personal agency in adolescents. Journal of Family Psychology.

[B100-behavsci-15-01154] Pad R. A., Okut H., Zackula R., Macaluso M., Huprich S. K. (2022). Understanding the relationship between personality pathology and attachment style in the context of the DSM-5 alternative model of personality disorders. Personality and Mental Health.

[B101-behavsci-15-01154] Pallini S., Morelli M., Chirumbolo A., Baiocco R., Laghi F., Eisenberg N. (2019). Attachment and attention problems: A meta-analysis. Clinical Psychology Review.

[B102-behavsci-15-01154] Paris J. (2004). Gender differences in personality traits and disorders. Current Psychiatry Reports.

[B103-behavsci-15-01154] Parker G., Roy K., Wilhelm K., Mitchell P., Austin M. P., Hadzi-Pavlovic D. (1999). An exploration of links between early parenting experiences and personality disorder type and disordered personality functioning. Journal of Personality Disorders.

[B104-behavsci-15-01154] Pereg D., Mikulincer M. (2004). Attachment style and the regulation of negative affect: Exploring individual differences in mood congruency effects on memory and judgment. Personality and Social Psychology Bulletin.

[B105-behavsci-15-01154] Perry J. C. (1990). Defense mechanism rating scales (DMRS).

[B106-behavsci-15-01154] Perry J. C., Cooper S. H. (1989). An empirical study of defense mechanisms: I. Clinical interview and life vignette ratings. Archives of General Psychiatry.

[B107-behavsci-15-01154] Ponsi M., Zeigler-Hill V., Shackelford T. (2017). Acting out (defense mechanism). Encyclopedia of personality and individual differences.

[B108-behavsci-15-01154] Remmers C., Bohn J., Hörz-Sagstetter S., Kampe L. (2023). Preliminary findings on the associations between defense mechanisms and implicit versus explicit negative affect. Psychoanalytic Psychology.

[B109-behavsci-15-01154] Richardson E., Beath A., Boag S. (2023). Default defenses: The character defenses of attachment-anxiety and attachment-avoidance. Current Psychology.

[B110-behavsci-15-01154] Ro E., Nuzum H., Clark L. A. (2017). Antagonism trait facets and comprehensive psychosocial disability: Comparing information across self, informant, and interviewer reports. Journal of Abnormal Psychology.

[B111-behavsci-15-01154] Rowe A. C., Gold E. R., Carnelley K. B. (2020). The effectiveness of attachment security priming in improving positive affect and reducing negative affect: A systematic review. International Journal of Environmental Research and Public Health.

[B112-behavsci-15-01154] Safran J. D., Muran J. C. (2000). Negotiating the therapeutic alliance: A relational treatment guide.

[B113-behavsci-15-01154] Santoro G., Midolo L. R., Costanzo A., Schimmenti A. (2021). The vulnerability of insecure minds: The mediating role of mentalization in the relationship between attachment styles and psychopathology. Bulletin of the Menninger Clinic.

[B114-behavsci-15-01154] Scharfe E., Bartholomew K. (1994). Reliability and stability of adult attachment patterns. Personal Relationships.

[B115-behavsci-15-01154] Schimmenti A. (2018). The trauma factor: Examining the relationships among different types of trauma, dissociation, and psychopathology. Journal of Trauma & Dissociation.

[B116-behavsci-15-01154] Schimmenti A., Caretti V. (2016). Linking the overwhelming with the unbearable: Developmental trauma, dissociation, and the disconnected self. Psychoanalytic Psychology.

[B117-behavsci-15-01154] Schimmenti A., Somer E., Regis M. (2019). Maladaptive daydreaming: Towards a nosological definition. Annales Médico-Psychologiques.

[B118-behavsci-15-01154] Schweizer K., Wang T., Ren X. (2025). Overestimation of internal consistency by coefficient omega in data giving rise to a centroid-like factor solution. Educational and Psychological Measurement.

[B119-behavsci-15-01154] Scott L. N., Levy K. N., Pincus A. L. (2009). Adult attachment, personality traits, and borderline personality disorder features in young adults. Journal of Personality Disorders.

[B120-behavsci-15-01154] Senad R. R. (2022). Gender differences in coping strategies (ego defense mechanisms) among professional and non-professional degree courses of students. International Journal of Health Sciences.

[B121-behavsci-15-01154] Shallcross A. J., Ford B. Q., Floerke V. A., Mauss I. B. (2013). Getting better with age: The relationship between age, acceptance, and negative affect. Journal of Personality and Social Psychology.

[B122-behavsci-15-01154] Siczek A., Cieciuch J. (2024). Pathological personality traits from ICD-11 and attachment—Comparison of 10 models of attachment dimensions. Patologiczne cechy osobowości z ICD-11 a przywiązanie—Porównanie 10 modeli wymiarów przywiązania. Psychiatria Polska.

[B123-behavsci-15-01154] Skabeikyte-Norkiene G., Sharp C., Kulesz P. A., Barkauskiene R. (2022). Personality pathology in adolescence: Relationship quality with parents and peers as predictors of the level of personality functioning. Borderline Personality Disorder and Emotion Dysregulation.

[B124-behavsci-15-01154] Slade A., Grienenberger J., Bernbach E., Levy D., Locker A. (2005). Maternal reflective functioning, attachment, and the transmission gap: A preliminary study. Attachment & Human Development.

[B125-behavsci-15-01154] Smith M. S., South S. C. (2024). Insecure attachment and personality pathology: Concurrent assessment and longitudinal modeling. Personality Disorders: Theory, Research, and Treatment.

[B126-behavsci-15-01154] Somer E. (2003). Prediction of abstinence from heroin addiction by childhood trauma, dissociation, and extent of psychosocial treatment. Addiction Research & Theory.

[B127-behavsci-15-01154] Spantidaki Kyriazi F. (2021). A motivational perspective of psychopathic traits: An investigation of motivated emotion regulation and motive dispositions.

[B128-behavsci-15-01154] Strandholm T., Kiviruusu O., Karlsson L., Miettunen J., Marttunen M. (2016). Defense mechanisms in adolescence as predictors of adult personality disorders. The Journal of Nervous and Mental Disease.

[B129-behavsci-15-01154] Tung Y. J., Lo K. K., Ho R. C., Tam W. S. W. (2018). Prevalence of depression among nursing students: A systematic review and meta-analysis. Nurse Education Today.

[B130-behavsci-15-01154] Ullman S. E., Najdowski C. J. (2009). Correlates of serious suicidal ideation and attempts in female adult sexual assault survivors. Suicide & Life-Threatening Behavior.

[B131-behavsci-15-01154] Vachon D. D., Miller J. A., Lynam D. R. (2019). Antagonism in psychopathy. The handbook of antagonism: Conceptualizations, assessment, consequences, and treatment of the low end of agreeableness.

[B132-behavsci-15-01154] Vaillant G. E. (1971). Theoretical hierarchy of adaptive ego mechanisms: A 30-year follow-up of 30 men selected for psychological health. Archives of General Psychiatry.

[B133-behavsci-15-01154] Vaillant G. E. (1992). Ego mechanisms of defense: A guide for clinicians and researchers.

[B134-behavsci-15-01154] Vaillant G. E., Kazdin A. E. (2000). Defense mechanisms. Encyclopedia of psychology.

[B135-behavsci-15-01154] Vondra J. I., Shaw D. S., Swearingen L., Cohen M., Owens E. B. (2001). Attachment stability and emotional and behavioral regulation from infancy to preschool age. Development and Psychopathology.

[B136-behavsci-15-01154] Wang X., Shao S., Cai Z., Ma C., Jia L., Blain S. D., Tan Y. (2024). Reciprocal effects between negative affect and emotion regulation in daily life. Behaviour Research and Therapy.

[B137-behavsci-15-01154] Watson D., Clark L. A. (1984). Negative affectivity: The disposition to experience aversive emotional states. Psychological Bulletin.

[B138-behavsci-15-01154] Watson D., Clark L. A. (2020). Personality traits as an organizing framework for personality pathology. Personality and Mental Health.

[B139-behavsci-15-01154] Yeomans F. E., Clarkin J. F., Kernberg O. F. (2015). Transference-focused psychotherapy for borderline personality disorder.

[B140-behavsci-15-01154] Zlotnick C., Shea M. T., Pearlstein T., Simpson E., Costello E., Begin A. (1996). The relationship between dissociative symptoms, alexithymia, impulsivity, sexual abuse, and self-mutilation. Comprehensive Psychiatry.

[B141-behavsci-15-01154] Zlotnick C., Shea M. T., Recupero P., Bidadi K., Pearlstein T., Brown P. (1997). Trauma, dissociation, impulsivity, and self-mutilation among substance abuse patients. American Journal of Orthopsychiatry.

